# Comparative efficacy of glucose-lowering drugs on liver steatosis as assessed by means of magnetic resonance imaging in patients with type 2 diabetes mellitus: systematic review and network meta-analysis

**DOI:** 10.1007/s42000-023-00493-z

**Published:** 2023-09-28

**Authors:** Konstantinos Malandris, Stylianos Papandreou, Ioannis Avgerinos, Thomas Karagiannis, Paschalis Paschos, Theodoros Michailidis, Aris Liakos, Eleni Bekiari, Emmanouil Sinakos, Apostolos Tsapas

**Affiliations:** 1 Clinical Research and Evidence-Based Medicine Unit, Second Medical Department, Aristotle University of Thessaloniki, Thessaloniki, Greece; 2 Diabetes Centre, Second Medical Department, Aristotle University of Thessaloniki, Konstantinoupoleos 49, 54642 Thessaloniki, Greece; 3 First Medical Department, “Papageorgiou” Hospital, Thessaloniki, Greece; 4 Fourth Medical Department, Hippokration General Hospital of Thessaloniki, Aristotle University of Thessaloniki, Thessaloniki, Greece; 5 Harris Manchester College, University of Oxford, Oxford, UK

**Keywords:** Type 2 diabetes, Liver steatosis, Systematic review, Network meta-analysis

## Abstract

**Purpose:**

To assess the comparative efficacy of glucose-lowering drugs on liver steatosis as assessed by means of magnetic resonance imaging (MRI) in patients with T2D.

**Methods:**

We searched several databases and grey literature sources. Eligible trials had at least 12 weeks of intervention, included patients with T2D, and assessed the efficacy of glucose-lowering drugs as monotherapies. The primary outcome of interest was absolute reduction in liver fat content (LFC), assessed by means of MRI. Secondary efficacy outcomes were reduction in visceral and subcutaneous adipose tissue. We performed random effects frequentist network meta-analyses to estimate mean differences (MDs) with 95% confidence intervals (CIs). We ranked treatments based on P-scores.

**Results:**

We included 29 trials with 1906 patients. Sodium-glucose cotransporter-2 (SGLT-2) inhibitors (P-score 0.84) and glucagon-like peptide-1 receptor agonists (GLP-1 RAs) (0.71) were the most efficacious in terms of liver fat content reduction. Among individual agents, empagliflozin was the most efficacious (0.86) and superior to pioglitazone (MD -5.7, 95% CI -11.2 to -0.3) (very low confidence). GLP-1 RAs had also the most favorable effects on visceral and subcutaneous adipose tissue.

**Conclusions:**

GLP-1 RAs and SGLT-2 inhibitors seem to be the most efficacious glucose-lowering drugs for liver steatosis in patients with T2D. Assessment of their efficacy on NAFLD in patients irrespective of presence of T2D is encouraged.

**Supplementary Information:**

The online version contains supplementary material available at 10.1007/s42000-023-00493-z.

## Introduction

Nonalcoholic fatty liver disease (NAFLD) is the most common hepatic disorder worldwide [1, 2]. It encompasses a wide spectrum of liver disease ranging from simple steatosis to nonalcoholic steatohepatitis (NASH), and ultimately liver cirrhosis [3]. Owing to their common pathophysiologic background, type 2 diabetes mellitus (T2D) and NAFLD are two closely related conditions that often co-exist [4]. The prevalence of NAFLD in patients with T2D is approximately 70% [5]. Furthermore, T2D is considered an important driver of NAFLD progression. Therefore, early recognition and management of NAFLD in patients with T2D is of major importance.

Liver biopsy is currently considered the reference standard for NAFLD diagnosis [3, 4]. Moreover, the Food and Drug Administration (FDA) and the European Medicines Agency (EMA) guidelines highlight the use of histological endpoints for conditional drug approval [6]. However, liver biopsy is an invasive procedure that may lead to severe complications, while it is impractical when it comes to patients’ follow-up. Moreover, its accuracy is undermined by sampling error and significant inter- and intra-observer variability concerning interpretation of the results [7]. As a result, novel imaging techniques are emerging as alternatives to liver biopsy for treatment response evaluation in patients with NAFLD. Among available modalities, magnetic resonance imaging (MRI)-derived proton density fat fraction (MRI-PDFF) is regarded as the most accurate for the assessment of liver fat content (LFC) [8]. Excessive fat accumulation can lead to liver necroinflammation and NASH progression. Recent meta-analyses support an association between a ≥ 30% relative decline in MRI-PDFF and histologic response in NAFLD [9, 10]. Consequently, the number of randomized controlled trials (RCTs) that use MRI techniques as an endpoint is greatly increasing.

Lifestyle modifications are the cornerstone for the management of NAFLD [3–5]. Up until recently, pioglitazone was the only glucose-lowering drug available for the management of NAFLD in patients with T2D [4]. Emerging evidence highlights the potentially beneficial role of other glucose-lowering drugs, including sodium-glucose cotransporter-2 inhibitors (SGLT-2i) and glucagon-like peptide-1 receptor agonists (GLP-1 RAs) in the management of NAFLD in the context of T2D [5, 11–13].

Given the increase of available medication options and the lack of head-to-head comparisons, we decided to perform a network meta-analysis in order to assess the comparative efficacy of available glucose-lowering drugs on liver steatosis in patients with T2D focusing on MRI-derived metrics. Previously published meta-analyses have addressed the efficacy of these agents in histological endpoints.

## Materials and methods

The protocol for this systematic review and network meta-analysis has been registered in PROSPERO (CRD42022381704). We report our systematic review and network meta-analysis in line with the PRISMA Extension Statement for Reporting Systematic Reviews Incorporating Network Meta-analyses of Health Care Interventions (Table S1) [14].

### Eligibility criteria

We included RCTs among adult patients with T2D that reported data on at least one outcome of interest and assessed glucose-lowering drugs currently approved by the FDA and/or EMA for the management of T2D. Eligible trials had a duration of treatment of at least 12 weeks and assessed the efficacy of drug monotherapies. Trials assessing agents that are not used in clinical practice, are no longer available, or have been withdrawn were not eligible for inclusion. Similarly, trials assessing non-pharmacologic interventions were excluded.

### Literature search

We searched Medline, Embase, and the Cochrane Register of Controlled Trials (CENTRAL) up to July 2022 without restrictions. We updated our search strategy up to June 2023 in order to include recently published RCTs. Our search strategy comprised free text terms and medical subject headings describing T2D, eligible interventions, and primary outcome of interest (Table S2). Moreover, ClinicalTrials.gov and European Union Drug Regulating Authorities Clinical Trials Database (EudraCT) registries were searched up to September 2022 for additional completed trials with available results. Furthermore, we searched conference proceedings of the American Association for the Study of Liver Diseases, the American Diabetes Association, the European Association for the Study of the Liver, and the European Association for the Study of Diabetes from 2016 to 2022.

### Study selection

Records from the electronic databases were imported into a literature review software (DistillerSR. Version 2.35). After duplicate removal, two reviewers working independently assessed record eligibility firstly at title and abstract level and subsequently in full text. Disagreements were arbitrated by a senior reviewer.

### Data extraction

Two independent reviewers performed data extraction using a predesigned form. Multiple reports for the same trial were collated into a single entry based on the trial’s registration number, title, and baseline characteristics. For trials assessing multiple eligible doses for a given agent, we combined data from approved doses into a single intervention group [15]. In the case of several time points, we extracted data for the longest duration of intervention for each outcome. Data extraction items included trial characteristics, participants’ baseline characteristics, and outcome data. When needed, we imputed data using appropriate methodology [16, 17]. The primary outcome of interest was the absolute change from baseline in liver fat content (LFC) measured by means of MRI [MRI-PDFF or MR-spectroscopy (MRS)]. Both imaging techniques measure LFC as a percentage through the fat and water signals acquired during a magnetic resonance examination [18]. Secondary outcomes of interest were change from baseline in visceral adipose tissue (VAT), change from baseline in subcutaneous adipose tissue (SAT), and change from baseline in VAT/SAT ratio. Both VAT and SAT were measured in square centimeters (cm^2^) by means of MRI.

### Risk of bias assessment

Risk of bias assessment was performed for all outcomes of interest by two independent reviewers with the revised Cochrane Risk of Bias tool (RoB) 2.0, assessing the following domains: randomization, deviations from intended interventions, missing outcome data, measurement of the outcome, and selection of reported results [19]. A trial was deemed at low risk of bias if all domains were at low risk. Trials were deemed at high risk of bias if at least one domain was at high risk or at least three domains aroused certain concerns. In any other case, a trial was considered to raise some concerns of bias.

### Transitivity assessment

Conducting a network meta-analysis entails the transitivity assumption among eligible comparisons [20]. We evaluated transitivity by taking into consideration the distribution of major effect modifiers across pairwise comparisons, including diabetes duration and baseline hemoglobin A_1C_, serum lipids, body mass index (BMI), and LFC.

### Data synthesis and analysis

We performed random effects, pairwise meta-analysis for each direct comparison with at least two trials. Subsequently, we performed frequentist random effects network meta-analysis and calculated mean differences (MDs) and 95% confidence intervals (CIs) for all outcomes of interest, assuming a common heterogeneity parameter across eligible comparisons [21, 22]. We ranked treatments by means of P-scores [23]. We evaluated statistical heterogeneity in the entire network based on the magnitude of the heterogeneity variance parameter (τ^2^), which derived from the network meta-analysis model. For our outcomes, we compared the estimated τ^2^ values with their expected values, as described by Turner et al.[24] We assessed inconsistency both locally with the side-splitting method and globally with the design-by-treatment model [25, 26].

For the primary outcome, eligible interventions were analyzed both as drug classes and as individual agents. For secondary outcomes and for all additional analysis, eligible interventions were analyzed as drug classes. We performed sensitivity analysis of trials with drug naïve or metformin monotherapy treated patients and trials that recruited patients with T2D and NAFLD at baseline. Moreover, we conducted sensitivity analyses including only trials at low RoB and trials where imputation methods were not performed for missing measures of dispersion. For all analyses, we used RevMan 5.4 and R statistical software.

### Certainty of evidence

We assessed the confidence of effect estimates for our primary outcome with the CINeMA (Confidence In the results from Network Meta-Analysis) methodological approach and web application. We took into consideration the following domains: within-study bias, across-study bias, indirectness, imprecision, heterogeneity, and incoherence [27, 28].

## Results

### Overview of trials

The study selection process is depicted in Fig. S1. After duplicate removal, we screened 2629 records and included 49 records for 29 RCTs with 1906 patients [29–57]. Trial and participant baseline characteristics are presented in Table S3. Overall, eligible RCTs assessed 14 interventions from eight different drug classes (DPP-4 inhibitors, GLP-1 RAs, GIP/GLP-1 RAs, basal insulins, metformin, pioglitazone, SGLT-2i, and sulphonylureas). Most trials (n = 21) were funded by the pharmaceutical industry. Sixteen trials [30–34, 36–38, 40, 41, 44, 45, 47, 50, 51, 57] with 943 patients were placebo-controlled, whereas two trials compared active interventions to standard of care [55, 56]. Three trials with 467 patients were multi-arm [35, 36, 52]. The majority of the trials were either double or single blinded. Most trials (n = 20) had a duration of intervention greater than or equal to 24 weeks. Sample size ranged from 12 to 296 patients.

Approximately 60% of the overall population were male (n = 1143). Patients’ mean age ranged from 43.1 to 65.6 years. Fifty-two percent of all patients were on a structured diet program or received dietary counseling, while 46% were on a structured exercise program or received some form of exercise counseling. Patients’ mean BMI at baseline ranged from 23.9 to 41.6 kg/m^2^. More than half of the included trials (n = 16) included mainly obese patients (BMI > 30.0 kg/m^2^). Mean HbA1c at baseline ranged from 6.3% to 9.1%. A total of 665 patients (34.8%) were either drug naïve or treated solely with metformin at enrolment. Among the remaining patients, the background glucose-lowering treatment varied, mainly comprising metformin with at least one more glucose-lowering agent.

### Risk of bias assessment

Risk of bias assessment for all outcomes is presented in Tables S4-S7. For the primary outcome, eight trials were at low risk and two trials to have some concerns of bias due to inadequate description of the randomization process and missing outcome data. The remaining trials were judged at high risk of bias because of suboptimal reporting of the analysis process and missing outcome data.

### Transitivity assessment

The available number of trials for each comparison was limited and, consequently, a comprehensive evaluation of transitivity was not possible. In order to assess the transitivity assumption, we compared potential effect modifiers across included trials. Patients’ mean duration of diabetes at enrolment was at least 3.0 years in all trials except one, which included solely patients with newly diagnosed T2D. Mean HbA1c at baseline was > 7.0% (53 mmol/mol) in the majority of trials, suggesting suboptimal glycemic control among patients. Moreover, most trials included middle-aged patients with comparable serum lipid profiles at enrolment. Across all trials, the median LFC at baseline was 16.15% (interquartile range, 13.1% to 21.6%), suggesting that the majority of patients at baseline had NAFLD.

### Pairwise meta-analyses

Results from pairwise meta-analyses are presented in Table S8. Sodium-glucose cotransporter-2 inhibitors (MD -3.06%, 95% CI -4.74 to -1.37) and GLP-1 RAs (-2.38%, -4.40 to -0.35) reduced LFC compared with placebo. Glucagon-like peptide-1 receptor agonists were superior to placebo in VAT (-23.4 cm^2^, -41.4 to -5.3) and SAT (-27.3 cm^2^, -38.0 to -16.7) reduction.

### Network meta-analyses

#### Liver fat content

**Fig. 1:**
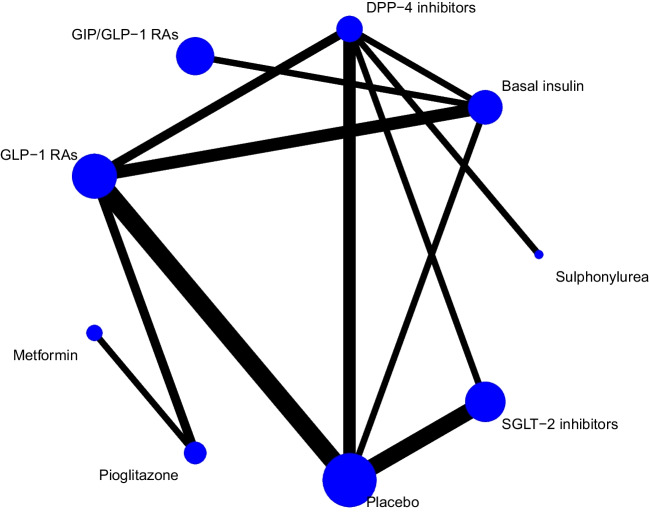
Network for change in liver fat content; drug classes. Each circle indicates a treatment node. Lines connecting two nodes represent direct comparisons between two treatments. The size of the nodes is proportional to the number of trials evaluating each treatment. The thickness of the lines is proportional to the number of trials directly comparing connected treatments. DPP-4 = dipeptidyl peptidase-4. GLP-1 = glucagon-like peptide-1. GIP = glucose-dependent insulinotropic polypeptide. SGLT-2 = sodium-glucose cotransporter-2. RA = receptor agonist

**Fig. 2:**
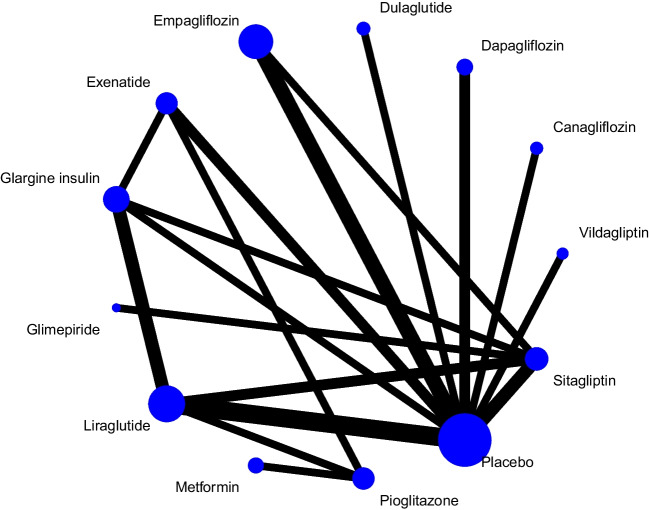
Network for change in liver fat content; agents. Each circle indicates a treatment node. Lines connecting two nodes represent direct comparisons between two treatments. The size of the nodes is proportional to the number of trials evaluating each treatment. The thickness of the lines is proportional to the number of trials directly comparing connected treatments

Network plots for change in LFC in terms of drug classes and individual agents are presented in Fig. 1 and Fig. 2, respectively. At drug class level, SGLT-2i (-3.27%, -4.99 to -1.56) and GLP-1 RAs (-2.22%, -3.87 to -0.57) reduced LFC compared to placebo. In comparisons between drug classes, GLP-1 RAs, SGLT-2i, and GIP/GLP-1 RAs were more efficacious compared to metformin, while compared to each other all three drug classes were equally efficacious (Table 1). Based on P-scores, GIP/GLP-1 RAs were ranked as the most efficacious treatment (P-score 0.87), followed by SGLT-2i (0.84) and GLP-1 RAs (0.71) (Table S9).

**Table 1:** Network meta-analysis results for liver fat content reduction (drug classes)

GIP/GLP-1 RAs								
-1.33 [ -6.96; 4.30]	SGLT-2 inhibitors							
-2.38 [ -7.66; 2.90]	-1.05 [ -3.38; 1.27]	GLP-1 RAs						
-3.44 [ -9.05; 2.17]	-2.11 [ -4.65; 0.43]	-1.06 [ -3.52; 1.41]	DPP-4 inhibitors					
-4.29 [ -8.92; 0.34]	-2.96 [ -6.15; 0.23]	-1.91 [ -4.44; 0.63]	-0.85 [ -4.02; 2.31]	Basal insulin				
-5.67 [-12.40; 1.07]	-4.34 [ -9.12; 0.45]	-3.28 [ -7.47; 0.90]	-2.23 [ -7.08; 2.63]	-1.38 [ -6.27; 3.52]	Pioglitazone			
-7.54 [-25.94; 10.86]	-6.21 [-23.92; 11.50]	-5.16 [-22.85; 12.54]	-4.10 [-21.62; 13.42]	-3.25 [-21.06; 14.56]	-1.87 [-20.06; 16.31]	Sulphonylurea		
**-9.03 [-17.23; -0.82]**	**-7.70 [-14.39; -1.00]**	**-6.64 [-12.92; -0.37]**	-5.59 [-12.33; 1.15]	-4.74 [-11.50; 2.03]	-3.36 [ -8.04; 1.32]	-1.49 [-20.26; 17.29]	Metformin	
-4.60 [-10.00; 0.79]	**-3.27 [ -4.99; -1.56]**	**-2.22 [ -3.87; -0.57]**	-1.16 [ -3.41; 1.08]	-0.31 [ -3.08; 2.46]	1.06 [ -3.43; 5.56]	2.94 [-14.73; 20.60]	4.42 [ -2.07; 10.91]	Placebo

Data are mean differences (95% CIs) of the column-defining treatment compared with the row-defining treatment. Negative values favor the column-defining treatment and positive values favor the row-defining treatment. Significant results are in bold. DPP-4 = dipeptidyl peptidase-4. *GIP* = glucose-dependent insulinotropic polypeptide. *GLP-1* = glucagon-like peptide-1. *SGLT-2* = sodium-glucose cotransporter-2. *RA* = receptor agonist

Among individual agents, only empagliflozin (-4.35%, -6.74 to -1.96) reduced LFC compared with placebo. Empagliflozin was also superior to pioglitazone (-5.78%, -11.21 to -0.34), metformin, insulin glargine, and sitagliptin (Table S10). There was no difference between canagliflozin, dapagliflozin, and empagliflozin. Empagliflozin was placed at the top of the hierarchy of competing treatments (0.86), followed by exenatide (0.77) and dulaglutide (0.73) (Table S11).

#### Visceral and subcutaneous adipose tissue

Network plots of trials assessing VAT and SAT reduction are presented in Fig. S2-S3. Compared to placebo, GLP-1 RAs reduced visceral (-25.0 cm^2^, -39.6 to -10.3) and subcutaneous ( -30.9 cm^2^, -44.6 to -17.2) adipose tissue. Glucagon-like peptide-1 receptor agonists and SGLT-2i were equally efficacious for both outcomes (Tables S12-S13). GLP-1 RAs were ranked as the best option for both outcomes (Tables S14-S15).

Data synthesis for VAT/SAT ratio was not feasible due to the limited number of trials reporting relevant data.

#### Additional analyses

All additional analyses were performed for the outcome of LFC reduction and for comparisons among drug classes. In sensitivity analysis of trials that recruited solely drug naïve or metformin monotherapy treated patients, only GLP-1 RAs reduced LFC compared with placebo (-4.54%, -7.56 to -1.51) (Table S16). Sensitivity analyses of trials that recruited patients with T2D and NAFLD and of trials at low risk of bias yielded similar results to our main analysis (Tables S17-S18). Results from additional analyses are presented in the supplementary appendix.

#### Heterogeneity and inconsistency and publication bias

For most networks, there was increased heterogeneity (Table S20). There was no evidence of inconsistency under the assumption of a full design-by-treatment interaction random effects model except for the VAT network (Table S21). Based on comparison-adjusted funnel plots, there was no evidence of small study effect bias.

#### Certainty of evidence

The confidence in estimates for reduction of LFC was low to very low across comparisons. This was mainly attributed to within-study bias and imprecision (Tables S22-S23).

## Discussion

### Summary of findings

The aim of this systematic review and network meta-analysis was to assess the comparative efficacy of glucose-lowering drugs on liver steatosis as assessed by means of MRI in patients with T2D. In terms of drug classes, GLP-1 RAs and SGLT-2 inhibitors were the most efficacious in reducing LFC based on MRI. Empagliflozin was ranked as the most efficacious glucose-lowering agent, followed by exenatide and dulaglutide. Tirzepatide, a novel agent recently licensed for the treatment of T2D, seems promising; nevertheless, results derive from a single trial and therefore firm statements regarding its effect on liver steatosis are rather challenging. Pioglitazone, a glucose-lowering agent with proven histologic efficacy in biopsy-confirmed NASH, performed poorly. The confidence in our estimates was low to very low. In terms of adipose tissue, either visceral or subcutaneous, GLP-1RAs were the most efficacious options.

Our results are generally in line with recently published guidelines supporting the use of GLP1-RAs and SGLT-2i in patients with T2D and NAFLD [5, 58]. Weight reduction and management of cardiovascular risk are the cornerstone for the management of NAFLD. Glucagon-like peptide-1 receptor agonists and SGLT-2i already have an established efficacy for weight reduction and management of aspects of metabolic syndrome [59, 60]. Our analysis corroborates findings from previous meta-analyses regarding the efficacy of GLP-1 RAs and SGLT-2 inhibitors on liver steatosis, as assessed by means of MRI [61, 62]. Given the fact that hepatic lipotoxocity is a major risk factor of fibrosis progression in NAFLD, our findings suggest that certain glucose-lowering drug classes and agents have the potential to reduce liver steatosis and possibly halt fibrosis in patients with T2D [8, 63]. Among existing glucose-lowering agents, pioglitazone is the most efficacious for NASH amelioration as assessed by means of liver biopsy. Discrepancies regarding the efficacy of pioglitazone on liver steatosis are probably attributable to the fact that we did not assess histological outcomes, for which the agent has proven efficacy, and that our population of interest comprised patients with T2D irrespective of the presence of NAFLD at baseline [4, 63].

In line with our findings, other meta-analyses highlight the efficacy of GLP-1RAs with regard to VAT and SAT reduction in patients with T2D [64]. Based on the ‘adipose tissue overflow’ hypothesis, fat storage begins primarily in the subcutaneous region. When the subcutaneous region exceeds its capacity, fat accumulates in deeper regions such as the viscera or liver [65]. These deeper fat deposits are more pathogenic, contributing to the inflammatory pathway related to NAFLD [66].

### Strengths and limitations

In comparison to other meta-analyses [63, 67, 68], we focused on MRI-derived metrics based on the need for noninvasive assessment of liver steatosis. Glucose-lowering agents are currently at the epicenter of the NASH clinical trial landscape. Following the limited number of clinical trials comparing active interventions, we provide preliminary comparative efficacy estimates among promising agents and drug classes. Furthermore, we assessed both interclass and intraclass differences among treatments and synthesized available evidence using robust methodology.

Certain limitations should be acknowledged. Most trials were at high risk of bias and the confidence in our estimates among comparisons was low to very low. We did not evaluate a variety of pertinent clinically important outcomes (i.e., histological, biochemical, and safety related outcomes). On the other hand, histological and biochemical parameters as well as the safety of glucose-lowering agents have been addressed in previous meta-analyses [60–63]. Focusing on the presence of T2D as an inclusion criterion meant that we had to exclude RCTs that assessed the efficacy of glucose-lowering agents on liver steatosis in patients with stablished NAFLD, regardless of the presence of T2D [8, 69]. Results from these trials highlight the beneficial role of semaglutide in the reduction of intrahepatic fat, although the effects on fibrosis were not significant. In addition, heterogeneity is always a concern in evidence synthesis, especially in the context of network meta-analysis, thus limiting the validity of results [20]. Moreover, individual agent analysis was based on a limited number of trials per comparison; thus, results should be interpreted with caution. Furthermore, although treatment ranking by means of either SUCRA or p scores seems attractive, it can sometimes be misleading. Estimates of ranking probabilities are closely related to network structure and number of trials per comparison. The same number of trials per comparison can lead to biased ranking estimates for a given network [70]. In our analysis, the GIP/GLP1-RA tirzepatide has a p score of 0.87, suggesting high probability of being the best for the reduction of LFC among drug classes, although it failed to outperform placebo. This phenomenon can be partially addressed by taking into consideration the certainty of evidence when interpreting synthesis results.

### Implication for practice and research

Our results provide supportive evidence on the use of GLP-1RAs and SGLT-2i as suggested by current practice guidelines [5, 58]. Among SGLT-2i, our results support the use of empagliflozin alongside semaglutide in patients with T2D and liver steatosis. Tirzepatide, a novel GIP/GLP1-RA, seems promising; however, its efficacy in terms of steatosis management remains to be established. As a result, future research should focus on the assessment of the above intervention in the context of NAFLD irrespective of the presence of diabetes. Furthermore, the efficacy of these interventions in patients with metabolic syndrome but without diabetes remains unclear. Whether glucose-lowering agents exert their beneficial effects on liver steatosis through body weight reduction is still a matter of controversy. In our study, drug classes with proven benefit in weight reduction were also the most efficacious in reducing liver steatosis, whereas treatment with pioglitazone, which has proven efficacy in histologic improvement of NASH but is also associated with an increased risk for weight gain, performed poorly. Further research should shed light on whether counterbalancing pioglitazone-induced weight gain by combination therapy with agents that induce weight loss could maximize benefits regarding liver steatosis. Nevertheless, several trials have demonstrated the beneficial effect of pioglitazone on NAFLD irrespective of the presence of diabetes [71, 72]. Moreover, the combined efficacy of GLP-1RAs and SGLT-2i in NAFLD remains unclear.

Biopsy is the reference standard for the diagnosis of NAFLD. However, it is an invasive procedure with inherent limitations that hamper the follow-up of patients in trials. Undoubtedly, noninvasive techniques are the future of research in the field of NAFLD. Among existing noninvasive candidates, MRI seems the most promising. Consequently, MRI-related outcomes should be taken into consideration in future trials. A more holistic approach including histological, MRI-related, biochemical, anthropometric, and safety outcomes could provide deeper insight.

## Conclusion

Our results suggest that GLP-1 RAs and SGLT-2 inhibitors are the most efficacious glucose-lowering drug classes for amelioration of steatosis assessed by MRI-derived metrics in patients with T2D. Excluding semaglutide, empagliflozin is probably the best option among the available glucose-lowering agents. Conclusions must be interpreted with caution, since confidence in our estimates is very low, and, as such, large-scale, high-quality RCTs with MRI-related outcomes are needed.

### Supplementary Information

Below is the link to the electronic supplementary material.

Supplementary file1 (DOCX 435 KB)

## Data Availability

Available on reasonable request from Dr Malandris.
