# Supplementary material for: Comparative efficacy of glucose-lowering drugs on liver steatosis as assessed by means of magnetic resonance imaging in patients with type 2 diabetes mellitus: systematic review and network meta-analysis
Source: Hormones (Athens). 2023 Sep 28;22(4):655–64. doi: 10.1007/s42000-023-00493-z (PMC10651545; doi:10.1007/s42000-023-00493-z)
Supplement: Supplementary file 1 — Supplementary file1 (DOCX 435 KB) [file 42000_2023_493_MOESM1_ESM.docx]

**Supplementary Appendix**

**Comparative efficacy of** **glucose-lowering drugs on liver steatosis as assessed by means of magnetic resonance imaging in patients with type 2 diabetes mellitus. Systematic review and network meta-analysis**

| **Table S1:** |  | **PRISMA NMA Checklist of Items to Include When Reporting A Systematic Review Involving a Network Meta-analysis** |  |
| --- | --- | --- | --- |
| **Section/Topic** | **Item #** | **Checklist Item** | **Reported on Page #** |
| **TITLE** |  |  |  |
| Title | 1 | Identify the report as a systematic review *incorporating a network meta-analysis (or related form of meta-analysis).* | ***1*** |
|  |  |  |  |
| **ABSTRACT** |  |  | ***3*** |
| Structured summary | 2 | Provide a structured summary including, as applicable:  **Background:** main objectives  **Methods:** data sources; study eligibility criteria, participants, and interventions; study appraisal; and *synthesis methods, such as network meta-analysis.*  **Results:** number of studies and participants identified; summary estimates with corresponding confidence/credible intervals; *treatment rankings may also be discussed. Authors may choose to summarize pairwise comparisons against a chosen treatment included in their analyses for brevity.*  **Discussion/Conclusions:** limitations; conclusions and implications of findings.  **Other:** primary source of funding; systematic review registration number with registry name. |  |
|  |  |  |  |
| **INTRODUCTION** |  |  |  |
| Rationale | 3 | Describe the rationale for the review in the context of what is already known*, including mention of why a network meta-analysis has been conducted.* | ***4*** |
| Objectives | 4 | Provide an explicit statement of questions being addressed, with reference to participants, interventions, comparisons, outcomes, and study design (PICOS). | 4-5 |
|  |  |  |  |
| **METHODS** |  |  |  |
| Protocol and registration | 5 | Indicate whether a review protocol exists and if and where it can be accessed (e.g., Web address); and, if available, provide registration information, including registration number. | 5 |
| Eligibility criteria | 6 | Specify study characteristics (e.g., PICOS, length of follow-up) and report characteristics (e.g., years considered, language, publication status) used as criteria for eligibility, giving rationale. *Clearly describe eligible treatments included in the treatment network, and note whether any have been clustered or merged into the same node (with justification).* | ***5*** |
| Information sources | 7 | Describe all information sources (e.g., databases with dates of coverage, contact with study authors to identify additional studies) in the search and date last searched. | 5 |
| Search | 8 | Present full electronic search strategy for at least one database, including any limits used, such that it could be repeated. | supp info |
| Study selection | 9 | State the process for selecting studies (i.e., screening, eligibility, included in systematic review, and, if applicable, included in the meta-analysis). | 5-6 |
| Data collection process | 10 | Describe method of data extraction from reports (e.g., piloted forms, independently, in duplicate) and any processes for obtaining and confirming data from investigators. | 6 |
| Data items | 11 | List and define all variables for which data were sought (e.g., PICOS, funding sources) and any assumptions and simplifications made. | 6 |
| **Geometry of the network** | **S1** | Describe methods used to explore the geometry of the treatment network under study and potential biases related to it. This should include how the evidence base has been graphically summarized for presentation, and what characteristics were compiled and used to describe the evidence base to readers. | ***supp info*** |
| Risk of bias within individual studies | 12 | Describe methods used for assessing risk of bias of individual studies (including specification of whether this was done at the study or outcome level), and how this information is to be used in any data synthesis. | 6 |
| Summary measures | 13 | State the principal summary measures (e.g., risk ratio, difference in means). *Also describe the use of additional summary measures assessed, such as treatment rankings and surface under the cumulative ranking curve (SUCRA) values, as well as modified approaches used to present summary findings from meta-analyses.* | 7 |
| Planned methods of analysis | 14 | Describe the methods of handling data and combining results of studies for each network meta-analysis. This should include, but not be limited to:   - *Handling of multi-arm trials;* - *Selection of variance structure;* - *Selection of prior distributions in Bayesian analyses; and* - *Assessment of model fit.* | 7 |
| **Assessment of Inconsistency** | **S2** | Describe the statistical methods used to evaluate the agreement of direct and indirect evidence in the treatment network(s) studied. Describe efforts taken to address its presence when found. | 7 |
| Risk of bias across studies | 15 | Specify any assessment of risk of bias that may affect the cumulative evidence (e.g., publication bias, selective reporting within studies). | 6 |
| Additional analyses | 16 | Describe methods of additional analyses if done, indicating which were pre-specified. This may include, but not be limited to, the following:   - Sensitivity or subgroup analyses; - Meta-regression analyses; - *Alternative formulations of the treatment network; and* - *Use of alternative prior distributions for Bayesian analyses (if applicable).* | 7 |
|  |  |  |  |
| **RESULTS†** |  |  |  |
| Study selection | 17 | Give numbers of studies screened, assessed for eligibility, and included in the review, with reasons for exclusions at each stage, ideally with a flow diagram. | 7, supp inf |
| **Presentation of network structure** | **S3** | Provide a network graph of the included studies to enable visualization of the geometry of the treatment network. | ***Fig1,2,supp info*** |
| **Summary of network geometry** | **S4** | Provide a brief overview of characteristics of the treatment network. This may include commentary on the abundance of trials and randomized patients for the different interventions and pairwise comparisons in the network, gaps of evidence in the treatment network, and potential biases reflected by the network structure. | ***7-8*** |
| Study characteristics | 18 | For each study, present characteristics for which data were extracted (e.g., study size, PICOS, follow-up period) and provide the citations. | 7-8, supp info |
| Risk of bias within studies | 19 | Present data on risk of bias of each study and, if available, any outcome level assessment. | 8, supp info |
| Results of individual studies | 20 | For all outcomes considered (benefits or harms), present, for each study: 1) simple summary data for each intervention group, and 2) effect estimates and confidence intervals. *Modified approaches may be needed to deal with information from larger networks.* | ***supp info*** |
| Synthesis of results | 21 | Present results of each meta-analysis done, including confidence/credible intervals. *In larger networks, authors may focus on comparisons versus a particular comparator (e.g. placebo or standard care), with full findings presented in an appendix. League tables and forest plots may be considered to summarize pairwise comparisons.* If additional summary measures were explored (such as treatment rankings), these should also be presented. | ***9-10, supp info*** |
| **Exploration for inconsistency** | **S5** | Describe results from investigations of inconsistency. This may include such information as measures of model fit to compare consistency and inconsistency models, *P* values from statistical tests, or summary of inconsistency estimates from different parts of the treatment network. | ***10, supp info*** |
| Risk of bias across studies | 22 | Present results of any assessment of risk of bias across studies for the evidence base being studied. | 8, supp info |
| Results of additional analyses | 23 | Give results of additional analyses, if done (e.g., sensitivity or subgroup analyses, meta-regression analyses*, alternative network geometries studied, alternative choice of prior distributions for Bayesian analyses,* and so forth). | ***10, supp info*** |
|  |  |  |  |
| **DISCUSSION** |  |  |  |
| Summary of evidence | 24 | Summarize the main findings, including the strength of evidence for each main outcome; consider their relevance to key groups (e.g., healthcare providers, users, and policy-makers). | 10 |
| Limitations | 25 | Discuss limitations at study and outcome level (e.g., risk of bias), and at review level (e.g., incomplete retrieval of identified research, reporting bias). *Comment on the validity of the assumptions, such as transitivity and consistency. Comment on any concerns regarding network geometry (e.g., avoidance of certain comparisons).* | 11-12 |
| Conclusions | 26 | Provide a general interpretation of the results in the context of other evidence, and implications for future research. | 13 |
|  |  |  |  |
| **FUNDING** |  |  |  |
| Funding | 27 | Describe sources of funding for the systematic review and other support (e.g., supply of data); role of funders for the systematic review. This should also include information regarding whether funding has been received from manufacturers of treatments in the network and/or whether some of the authors are content experts with professional conflicts of interest that could affect use of treatments in the network. | ***2*** |

| **Table S2: Search strategy** |
| --- |
| **Medline via PubMed** |
| 1. "liver"[tiab] AND ("fatty"[tiab] OR "steatosis"[tiab] OR "steatoses"[tiab]) |
| 1. "NAFLD"[tiab] |
| 1. non alcoholic fatty liver disease[MeSH Terms] |
| 1. nafld OR fatty liver OR nash |
| 1. "hepatic steatosis" |
| 1. "Diabetes Mellitus, Type 2"[Mesh] |
| 1. NIDDM |
| 1. (Non insulin* AND depe*) OR (Noninsulin* AND depe*) OR (Non insulin depe*) (Type II diabet*) OR (Type 2 diabet*) OR (diabet* type 2) OR (diabet* type II) |
| 1. 1-8/ OR |
| 1. (randomized controlled trial[pt] OR controlled clinical trial[pt] OR randomized[tiab] OR placebo[tiab] OR drug therapy[sh] OR randomly[tiab] OR trial[tiab] OR groups[tiab] NOT (animals [mh] NOT humans [mh])) |
| 1. "glucagon-like peptide-1 receptor agonists" OR "GLP-1 receptor agonists" |
| 1. GLP1 OR GLP-1 |
| 1. "glucagon like peptide 1 receptor/agonists"[MeSH Terms] |
| 1. Liraglutide |
| 1. "Liraglutide"[Mesh] |
| 1. Lixisenatide |
| 1. "lixisenatide"[Supplementary Concept] |
| 1. Dulaglutide |
| 1. "dulaglutide"[Supplementary Concept] |
| 1. Exenatide |
| 1. "exenatide"[Supplementary Concept] |
| 1. Semaglutide |
| 1. "semaglutide"[Supplementary Concept] |
| 1. Tirzepatide |
| 1. "SGLT-2 inhibitors" OR "SGLT2" |
| 1. "sodium-glucose cotransporter-2 inhibitors" |
| 1. canagliflozin[MeSH Terms] |
| 1. canagliflozin |
| 1. "empagliflozin"[Supplementary Concept] |
| 1. Empagliflozin |
| 1. Dapagliflozin |
| 1. Ertugliflozin |
| 1. Sotagliflozin |
| 1. Metformin |
| 1. "Metformin"[Mesh] |
| 1. Pramlintide |
| 1. glimepiride |
| 1. "glimepiride"[Supplementary Concept] |
| 1. gliclazide |
| 1. "Gliclazide"[Mesh] |
| 1. glipizide |
| 1. "Glipizide"[Mesh] |
| 1. glyburide |
| 1. glibenclamide |
| 1. "Glyburide"[Mesh] |
| 1. DPP4 OR DPP-4 |
| 1. alogliptin |
| 1. "alogliptin"[Supplementary Concept] |
| 1. Linagliptin |
| 1. "Linagliptin"[Mesh] |
| 1. saxagliptin |
| 1. "saxagliptin"[Supplementary Concept] |
| 1. sitagliptin |
| 1. "Sitagliptin Phosphate"[Mesh] |
| 1. vildagliptin |
| 1. "vildagliptin"[Supplementary Concept] |
| 1. Pioglitazone |
| 1. "pioglitazone"[Supplementary Concept] |
| 1. Acarbose |
| 1. "Acarbose"[Mesh] |
| 1. miglitol |
| 1. "miglitol"[Supplementary Concept] |
| 1. nateglinide |
| 1. "nateglinide"[Supplementary Concept] |
| 1. repaglinide |
| 1. "repaglinide"[Supplementary Concept] |
| 1. insulin degludec |
| 1. "insulin degludec, insulin aspart drug combination"[Supplementary Concept] |
| 1. "insulin degludec"[Supplementary Concept] |
| 1. insulin detemir |
| 1. "Insulin Detemir"[Mesh] |
| 1. insulin glargine |
| 1. "Insulin Glargine"[Mesh] |
| 1. "LY2963016 insulin glargine"[Supplementary Concept] |
| 1. insulin aspart |
| 1. "Insulin Aspart"[Mesh] OR "insulin aspart, insulin aspart protamine drug combination 30:70"[Supplementary Concept] |
| 1. "insulin degludec, insulin aspart drug combination"[Supplementary Concept] |
| 1. insulin glulisine |
| 1. "insulin glulisine"[Supplementary Concept] |
| 1. insulin lispro |
| 1. "Insulin Lispro"[Mesh] |
| 1. "isophane insulin, insulin lispro drug combination 50:50"[Supplementary Concept] |
| 1. "insulin lispro, isophane insulin lispro drug combination (25:75)"[Supplementary Concept] |
| 1. insulin isophane |
| 1. insulin NPH |
| 1. insulin neutral protamine Hagedorn |
| 1. "Insulin, Isophane"[Mesh] |
| 1. insulin regular |
| 1. insulin human |
| 1. "Insulin, Regular, Human"[Mesh] |
| 1. MYL-1501D |
| 1. MYL1501D |
| 1. LY2963016 |
| 1. Humulin 70/30 |
| 1. "bile acid sequestrant" or "cholestyramine" or "colestimide" or "colestipol" or "colesevelam" |
| 1. colesevelam[MeSH Terms] |
| 1. 11-96/ OR |
| 1. MRI OR MRS OR MRE OR MRIPDFF OR MRI-PDFF |
| 1. magnetic resonance imaging [tiab] |
| 1. "magnetic resonance imaging"[MeSH Terms] |
| 1. magnetic resonance[tiab] |
| 1. magne* AND resona* |
| 1. 98-102/ OR |
| 1. 9 AND 10 AND 97 AND 103 |

**Figure S1:** Flow diagram

ClinicalTrials.gov, EudraCT, conference proccedings

(n = 4)

Electronic Databases

(Medline, Embase, CENTRAL)

(n = 2629)

## Identification

Records after duplicate removal
(n = 2184)

## Screening

Records screened
(n = 2184)

Records excluded
(n = 1966)

Full-text articles assessed for eligibility
(n =218)

Full-text articles excluded, with reasons (n =169)

Wrong outcomes (n = 43)

Wrong patient population (n = 44)

Wrong study design (n = 10)

Wrong intervention (n = 11)

Wrong comparator (n = 8)

Wrong treatment duration (n = 1)

Additional duplicates (n = 52)

## Eligibility

## Included

49 records for 29 trials included in quantitative synthesis (meta-analysis)

| **Table S3:** Baseline characteristics of included trials | | | | | | | | | | | | | | | |
| --- | --- | --- | --- | --- | --- | --- | --- | --- | --- | --- | --- | --- | --- | --- | --- |
| Author, Year | NCT | Background Treatment | Treatment arms | Number of patients | Males (%) | Mean Age | Mean  BMI | Mean Body Weight (kg) | Mean AST (U/L) | Mean ALT  (U/L) | Mean Diabetes  Duration | Mean  HBA1c  % (mmol/mol) | Mean  LDL  (mg/dl) | Mean  TG  (mg/dl) | Mean  LFC^4^  (%) |
| Bi 2014 | NCT01147627 | Drug naive | Exenatide 20mcg daily | 11 | 63.6 | 50.8 | 25.1 | 71.1 | 26.3 | 30.7 | NR | 8.6 (70) | 116.0 | 150.6 | 27.4 |
|  |  |  | Pioglitazone 45mg daily | 11 | 36.4 | 51.0 | 23.9 | 61.3 | 23.0 | 21.6 | NR | 8.3 (67) | 123.7 | 115.2 | 20.2 |
| Bizino 2019 | NCT01761318 | Metformin plus other drug(s) | Liraglutide 1.8mg daily | 23 | 61.0 | 60.0 | 32.6 | 98.0 | 31.0 | 15.0 | 11.0 | 8.4 (68) | 100.5 | 194.9 | 18.1 |
|  |  |  | Placebo | 26 | 58.0 | 59.0 | 31.6 | 94.0 | 35.0 | 13.0 | 11.0 | 8.2 (66) | 96.7 | 186.0 | 18.4 |
| Bolinder 2012 | NCT00855166 | Metformin monotherapy | Dapagliflozin 10mg daily | 38 | NR | NR | NR | NR | NR | NR | NR | NR | NR | NR | 13.5 |
|  |  |  | Placebo | 42 | NR | NR | NR | NR | NR | NR | NR | NR | NR | NR | 11.5 |
| Cusi 2019 | NCT02009488 | Metformin plus other drug(s) | Canagliflozin 300mg daily | 26 | 62.0 | 58.0 | 32.2 | 94.5 | 22.0 | 23.0 | NR | 7.6 (60) | NR | NR | 9.8 |
|  |  |  | Placebo | 30 | 70.0 | 58.0 | 31.0 | 92.5 | 27.0 | 35.0 | NR | 7.7 (61) | NR | NR | 14.8 |
| Eriksson 2018 | NCT02279407 | Metformin plus other drug(s) | Dapagliflozin 10mg daily | 21 | 76.2 | 65.0 | 30.5 | 90.2 | 31.2 | 40.2 | 6.7 | 7.4 (57) | 109.4 | 178.0 | 17.3 |
|  |  |  | Placebo | 21 | 81.0 | 65.6 | 30.3 | 93.0 | 29.4 | 34.2 | 6.5 | 7.4 (57) | 98.2 | 169.2 | 15.1 |
| Gaborit 2021 | NCT03118336 | Metformin plus other drug(s) | Empagliflozin 10mg daily | 26 | 38.0 | 57.0 | 33.6 | 92.3 | 25.3 | 32.3 | 10.9 | 8.2 (66) | 83.0 | 146.3 | 25.4 |
|  |  |  | Placebo | 25 | 40.0 | 58.6 | 34.7 | 93.7 | 25.0 | 36.7 | 11.0 | 8.1 (65) | 92.0 | 160.0 | 35.0 |
| Gastaldelli 2022 | NCT03882970 | Metformin plus other drug(s) | Degludec insulin | 74 | 54.0 | 56.5 | 33.0 | 91.2 | 21.1 | 27.4 | 7.0 | 8.1 (65) | 102.5 | 213.8 | 16.6 |
|  |  |  | Tirzepatide 5mg weekly | 71 | 62.0 | 56.5 | 34.5 | 98.0 | 22.8 | 32.3 | 7.9 | 8.3 (67) | 93.6 | 195.3 | 14.9 |
|  |  |  | Tirzepatide 10mg weekly | 79 | 52.0 | 55.6 | 33.1 | 93.1 | 21.8 | 29.5 | 9.4 | 8.4 (68) | 104.1 | 257.4 | 14.8 |
|  |  |  | Tirzepatide 15mg weekly | 72 | 65.0 | 56.5 | 33.4 | 95.6 | 22.5 | 30.0 | 8.7 | 8.2 (66) | 95.8 | 194.2 | 16.7 |
| Guo 2020 | ChiCTR2000035091 | Metformin monotherapy | Liraglutide 1.8mg daily | 32 | 52.0 | 53.1 | 29.2 | 84.3 | 29.6 | 33.2 | NR | 7.4 (57) | 131.5 | 168.3 | 25.0 |
|  |  |  | Glargine insulin | 32 | 60.0 | 52.0 | 28.3 | 83.8 | 27.9 | 31.5 | NR | 7.4 (57) | 116.0 | 194.9 | 25.8 |
|  |  |  | Placebo | 32 | 67.0 | 52.6 | 28.6 | 82.2 | 28.1 | 30.5 | NR | 7.4 (57) | 131.5 | 168.3 | 25.0 |
| Guzman 2017 | NCT02111096 | Metformin plus other drug(s) | Sitagliptin 100mg daily | 41 | 75.6 | 57.1 | 31.8 | 94.0 | 26.0 | 31.6 | 10.9 | 8.3 (67) | 98.6 | 178.0 | 14.8 |
|  |  |  | Placebo | 68 | 54.4 | 57.8 | 31.2 | 85.7 | 20.1 | 23.9 | 10.2 | 8.3 (67) | 100.9 | 165.6 | 11.5 |
| Harreiter 2021 | NR | Metformin plus other drug(s)^1^ | Exenatide 2mg weekly | 16 | 62.6 | 59.4 | 31.9 | 99.1 | 30.2 | 40.0 | 7.3 | 7.8 (62) | 85.1 | 132.9 | 12.9 |
|  |  |  | Placebo | 14 | 71.4 | 60.9 | 30.7 | 93.5 | 34.4 | 47.2 | 5.8 | 7.3 (56) | 92.8 | 159.4 | 13.1 |
| Hiruma 2023 | UMIN000026791 | Metformin plus other drug(s) | Empagliflozin 10mg daily | 23 | 73.9 | 52.4 | 30.6 | 85.5 | 37.0 | 59.2 | 4.6 | 7.1 (54) | 134.4 | NR | 19.9 |
|  |  |  | Sitagliptin 100mg daily | 19 | 68.4 | 50.0 | 28.6 | 80.1 | 37.7 | 60.6 | 3.3 | 7.2 (55) | 139.2 | NR | 22.1 |
| Jonker 2010 | NR | glimepiride monotherapy | Metformin 2000mg daily | 39 | 100.0 | 56.4 | 29.3 | NR | NR | NR | 3.0 | 7.0 (53) | 112.1 | 174.2 | 11.8 |
|  |  |  | Pioglitazone 30mg daily | 39 | 100.0 | 56.8 | 28.2 | NR | NR | NR | 4.3 | 7.1 (54) | 96.7 | 171.2 | 8.6 |
| Joy 2017 | NCT01260246 | Metformin and/or sulfonylurea | Sitagliptin 100mg daily | 6 | 50.0 | 56.7 | 35.9 | 100.4 | 44.0 | 72.0 | 11.5 | 7.9 (63) | 54.9 | 248.0 | 19.0 |
|  |  |  | Placebo | 6 | 33.0 | 54.7 | 37.4 | 105.8 | 39.0 | 46.0 | 5.7 | 8.2 (66) | 62.3 | 206.4 | 21.9 |
| Kahl 2020 | NCT02637973 | Drug naïve | Empagliflozin 25mg daily | 42 | 69.0 | 62.7 | 32.1 | 94.7 | 25.2 | 32.4 | 3.0 | 6.8 (51) | 133.0 | 161.0 | 9.6 |
|  |  |  | Placebo | 42 | 69.0 | 61.5 | 32.4 | 98.0 | 25.8 | 37.2 | 3.3 | 6.7 (50) | 120.0 | 178.3 | 11.3 |
| Kato 2015 | UMIN000013356 | Drug naïve  or Metformin monotherapy | Glimepiride 1mg daily | 10 | 50.0 | 53.0 | 28.0 | NR | NR | NR | NR | 7.2 (55) | 118.7 | 150.3 | 22.1 |
|  |  |  | Sitagliptin 50mg daily | 10 | 60.0 | 62.7 | 27.6 | NR | NR | NR | NR | 7.2 (55) | 131.0 | 154.0 | 26.7 |
| Liu 2020 | NCT02303730 | Drug naïve | Exenatide 20mcg daily | 35 | 54.3 | 47.6 | 28.5 | 79.3 | 31.3 | 42.7 | 0.3 | 8.3 (67) | 116.4 | 178.0 | 42.2 |
|  |  |  | Glargine insulin | 36 | 52.8 | 50.6 | 27.8 | 77.6 | 25.1 | 32.8 | 0.5 | 8.6 (70) | 108.7 | 213.5 | 35.5 |
| Macauley 2015 | NCT01356381 | Metformin monotherapy | Vildagliptin 100mg daily | 22 | NR | 65.2 | 29.4 | 83.0 | NR | 27.2 | NR | 6.5 (48) | NR | 132.9 | 7.3 |
|  |  |  | Placebo | 22 | NR | 58.9 | 31.1 | 91.8 | NR | 29.6 | NR | 6.4 (46) | NR | 124.0 | 5.4 |
| Matikainen 2018 | NCT02765399 | Metformin monotherapy | Liraglutide 1.8mg daily | 16 | 86.7 | 62.0 | 31.8 | 98.6 | NR | NR | 7.8 | 7.0 (53) | 81.2 | 150.9 | 14.8 |
|  |  |  | Placebo | 7 | 42.9 | 63.0 | 33.0 | 92.0 | NR | NR | 5.9 | 6.3 (45) | 96.7 | 124.0 | 16.1 |
| Ridderstråle 2014 | NCT01167881 | Metformin monotherapy | Empagliflozin 25mg daily | 51 | 47.1 | 57.6 | 31.5 | 85.2 | NR | NR | NR | NR | NR | NR | NR |
|  |  |  | Glimepiride  1-4mg daily | 40 | 40.0 | 53.2 | 32.3 | 84.9 | NR | NR | NR | NR | NR | NR | NR |
| Sathyanarayana 2011 | NCT01432405 | Metformin plus other drug(s)^2^ | Exenatide 20mcg daily | 11 | NR | NR | 34.1 | 95.5 | 25.0 | 35.0 | NR | 8.1 (65) | 118.0 | 136.0 | 12.1 |
|  |  |  | Placebo | 10 | NR | NR | 29.7 | 93.1 | 20.0 | 25.0 | NR | 8.3 (67) | 96.0 | 192.0 | 11.0 |
| Smits 2016 | NCT01744236 | Metformin and/or sulfonylurea | Liraglutide 1.8mg daily | 17 | 70.6 | 60.8 | 32.8 | 103.2 | 24.2 | 28.9 | 7.9 | 7.4 (57) | NR | NR | 20.9 |
|  |  |  | Sitagliptin 100mg daily | 18 | 82.4 | 61.5 | 31.4 | 98.5 | 22.8 | 28.9 | 8.5 | 7.1 (54) | NR | NR | 23.9 |
| Tang 2015 | NCT01399645 | Metformin plus other drug(s) | Liraglutide 1.8mg daily | 18 | 61.1 | 60.7 | 31.3 | 87.4 | 26.8 | 31.2 | NR | 7.6 (60) | 65.7 | 168.3 | 13.9 |
|  |  |  | Glargine insulin | 17 | 64.7 | 60.4 | 31.2 | 87.1 | 27.9 | 30.4 | NR | 7.9 (63) | 65.7 | 124.0 | 13.8 |
| Van Eyk 2019 | NCT02660047 | Metformin plus other drug(s) | Liraglutide 1.8mg daily | 22 | 36.0 | 55.0 | 30.4 | 81.9 | NR | NR | 19.0 | 8.1 (65) | 77.3 | 137.3 | 6.9 |
|  |  |  | Placebo | 25 | 44.0 | 55.0 | 28.6 | 77.8 | NR | NR | 17.0 | 8.6 (70) | 85.5 | 184.2 | 11.8 |
| Vanderheiden 2016 | NCT01505673 | Metformin plus other drug(s) | Liraglutide 1.8mg daily | 35 | 34.3 | 52.8 | 40.7 | 114.6 | NR | NR | 17.0 | 9.0 (75) | NR | NR | 15.7 |
|  |  |  | Placebo | 36 | 38.9 | 55.5 | 41.6 | 116.1 | NR | NR | 19.3 | 8.9 (74) | NR | NR | 10.9 |
| Yan 2019 | NCT02147925 | Metformin monotherapy | Liraglutide 1.8mg daily | 24 | 70.8 | 43.1 | 30.1 | 86.6 | 31.1 | 43.2 | 3.3 | 7.8 (62) | 104.4 | 203.7 | 15.4 |
|  |  |  | Glargine insulin | 24 | 58.3 | 45.6 | 29.6 | 85.6 | 33.2 | 39.5 | 5.8 | 7.7 (61) | 100.5 | 256.9 | 14.9 |
|  |  |  | Sitagliptin 100mg daily | 27 | 77.8 | 45.7 | 29.7 | 88.2 | 34.4 | 46.0 | 4.3 | 7.6 (60) | 119.9 | 230.3 | 15.5 |
| Zhang 2020 | NR | Drug naïve | Liraglutide 1.2mg daily | 30 | 43.3 | 50.2 | 27.6 | 79.3 | 33.1 | 30.0 | NR | 8.1 (65) | 127.6 | 217.9 | 24.1 |
|  |  |  | Pioglitazone 30mg daily | 30 | 50.0 | 51.5 | 27.1 | 78.0 | 33.1 | 36.6 | NR | 8.1 (65) | 127.6 | 217.9 | 23.9 |
| Kuchay 2020 | NCT03590626 | Metformin plus other drug(s)^3^ | Dulaglutide 1.5mg weekly | 32 | 72.0 | 46.6 | 29.6 | 85.8 | 49.9 | 70.1 | 4.9 | 8.4 (68) | 108.2 | 221.4 | 17.9 |
|  |  |  | Placebo | 32 | 69.0 | 48.1 | 29.9 | 83.7 | 46.1 | 68.1 | 5.7 | 8.4 (68) | 104.4 | 194.8 | 17.1 |
| Kuchay 2018 | NCT02686476 | Metformin plus other drug(s)^3^ | Empagliflozin 10mg daily | 22 | 58.9 | 50.7 | 30.0 | 80.8 | 44.6 | 64.3 | 6.6 | 9.0 (75) | 112.0 | 201.0 | 16.2 |
|  |  |  | Placebo | 20 | 60.0 | 49.1 | 29.4 | 81.2 | 45.3 | 65.3 | 6.8 | 9.1 (76) | 114.0 | 212.0 | 16.4 |
| Elhini 2022 | NCT04910178 | Metformin plus other drug(s) | Empagliflozin 25mg daily | 80 | 33.7 | 47.7 | 32.6 | NR | 29.5 | 28.7 | NR | 8.9 (74) | 152.4 | 191.0 | 21.5 |
|  |  |  | Placebo | 80 | 31.2 | 47.3 | 32.0 | NR | 25.8 | 26.0 | NR | 7.9 (63) | 93.5 | 140.3 | 19.9 |
| NR=Not reported, T2DM=Type 2 Diabetes Mellitus, AST=Aspartate transaminase, ALT=Alanine transaminase, HbA1c=Hemoglobin A1c, LDL=Low density lipoprotein, TG=Triglycerides, LFC=Liver fat content, BMI=Body mass index,  ^1^ Patients were randomized to exenatide plus dapagliflozin versus dapagliflozin plus placebo  ^2^ Patients were randomized to exenatide plus pioglitazone versus pioglitazone  ^3^ Patients were randomized to active intervention vs standard of care without active intervention  ^4^ LFC was assessed by either Magnetic resonance imaging derived proton density fat fraction (MRI-PDFF) or Proton magnetic resonance spectroscopy (H-MRS) | | | | | | | | | | | | | | | |

| **Table S4:** Risk of bias assessment for change in Liver fat content | | | | | | |
| --- | --- | --- | --- | --- | --- | --- |
| **Study** | **Randomization process** | **Deviations from intended interventions** | **Missing outcome data** | **Measurement of the outcome** | **Selection of reported results** | **Overall bias** |
| Bi 2014 | Low | Some concerns | High | Low | Low | High |
| Bizino 2019 | Low | Low | Low | Low | Low | Low |
| Bolinder 2012 | Low | Low | Some concerns | Low | Low | Some concerns |
| Cusi 2019 | Low | High | Low | Low | Low | High |
| Eriksson 2018 | Low | High | Low | Low | Low | High |
| Gaborit 2021 | Low | Low | Low | Low | Low | Low |
| Gastaldelli 2022 | Low | Low | Low | Low | Low | Low |
| Guo 2020 | Low | High | Low | Low | Low | High |
| Harreiter 2021 | Low | Low | Low | Low | Low | Low |
| Hiruma 2023 | Low | Low | High | Low | Low | High |
| Jonker 2010 | Some concerns | High | Low | Low | Low | High |
| Joy 2017 | Low | Low | Low | Low | Low | Low |
| Kahl 2020 | Low | Low | High | Low | Low | High |
| Kato 2015 | Some concerns | High | High | Low | Low | High |
| Liu 2020 | Low | Low | Low | Low | Low | Low |
| Macauley 2015 | Some concerns | High | Low | Low | Low | High |
| Guzman 2017 | Low | Low | High | Low | Low | High |
| Sathyanarayana 2011 | Low | High | Low | Low | Low | High |
| Smits 2016 | Low | High | Low | Low | Low | High |
| Tang 2015 | Low | Low | High | Low | Low | High |
| Van Eyk 2019 | Some concerns | Low | Low | Low | Low | Some concerns |
| Vanderheiden 2016 | Low | Low | Low | Low | Low | Low |
| Yan 2019 | Low | Low | Low | Low | Low | Low |
| Zhang 2020 | Some concerns | High | High | Low | Low | High |
| Matikainen 2018 | Some concerns | High | Low | Low | Low | High |
| Kuchay 2020 | Low | High | High | Low | Low | High |
| Kuchay 2018 | Low | High | Low | Low | Low | High |
| Elhini 2022 | Some concerns | High | Low | Low | Low | High |

| **Table S5:** Risk of bias assessment for change in Subcutaneous Adipose Tissue | | | | | | |
| --- | --- | --- | --- | --- | --- | --- |
| **Study** | **Randomization process** | **Deviations from intended interventions** | **Missing outcome data** | **Measurement of the outcome** | **Selection of reported results** | **Overall bias** |
| Bi 2014 | Low | Some concerns | High | Low | Low | High |
| Bizino 2019 | Low | Low | Low | Low | Low | Low |
| Gaborit 2021 | Low | Low | Low | Low | Low | Low |
| Guo 2020 | Low | High | Low | Low | Low | High |
| Liu 2020 | Low | Low | Low | Low | Low | Low |
| Van Eyk 2019 | Some concerns | Low | Low | Low | Low | Some concerns |
| Vanderheiden 2016 | Low | Low | Low | Low | Low | Low |
| Yan 2019 | Low | Low | Low | Low | Low | Low |
| Ridderstråle 2014 | Low | Low | Low | Low | Low | Low |
| Matikainen 2018 | Some concerns | High | Low | Low | Low | High |

| **Table S6:** Risk of bias assessment for change in Visceral Adipose Tissue | | | | | | |
| --- | --- | --- | --- | --- | --- | --- |
| **Study** | **Randomization process** | **Deviations from intended interventions** | **Missing outcome data** | **Measurement of the outcome** | **Selection of reported results** | **Overall bias** |
| Bi 2014 | Low | Some concerns | High | Low | Low | High |
| Bizino 2019 | Low | Low | Low | Low | Low | Low |
| Gaborit 2021 | Low | Low | Low | Low | Low | Low |
| Guo 2020 | Low | High | Low | Low | Low | High |
| Liu 2020 | Low | Low | Low | Low | Low | Low |
| Van Eyk 2019 | Some concerns | Low | Low | Low | Low | Some concerns |
| Vanderheiden 2016 | Low | Low | Low | Low | Low | Low |
| Yan 2019 | Low | Low | Low | Low | Low | Low |
| Ridderstråle 2014 | Low | Low | Low | Low | Low | Low |

| **Table S7:** Risk of bias assessment for change in VAT/SAT ratio | | | | | | |
| --- | --- | --- | --- | --- | --- | --- |
| **Study** | **Randomization process** | **Deviations from intended interventions** | **Missing outcome data** | **Measurement of the outcome** | **Selection of reported results** | **Overall bias** |
| Bolinder 2012 | Low | Low | Some concerns | Low | Low | Some concerns |
| Gastaldelli 2022 | Low | Low | Low | Low | Low | Low |
| Liu 2020 | Low | Low | Low | Low | Low | Low |
| Ridderstråle 2014 | Low | Low | Low | Low | Low | Low |

| **Table S8**: Results from pairwise meta-analyses | | | | | |
| --- | --- | --- | --- | --- | --- |
|  | Comparator | Number of Trials | Effect Estimate  MD | 95% Confidence Interval | Heterogeneity I^2^  (%) |
| **Absolute reduction in Liver Fat Content (LFC) (Drug Classes)** | | | | | |
| GLP-1 R agonists | Placebo | 9 | -2.38 | [-4.40, -0.35] | 79 |
| GLP-1 R agonists | Pioglitazone | 2 | -3.20 | [-7.10, 0.71] | 20 |
| GLP-1 R agonists | DPP4 inh | 2 | 0.14 | [-1.98, 2.26] | 0 |
| GLP-1 R agonists | Basal Insulin | 4 | -2.39 | [-5.29, 0.50] | 72 |
| SGLT-2 inh | Placebo | 7 | -3.06 | [-4.74, -1.37] | 77 |
| DPP4 inh | Placebo | 2 | -0.30 | [-4.30, 3.71] | 40 |
| **Absolute reduction in Liver Fat Content (LFC) (Agents)** | | | | | |
| Liraglutide | Placebo | 6 | -2.35 | [-5.03, 0.32] | 86 |
| Liraglutide | Glargine Insulin | 3 | -1.56 | [-4.47, 1.34] | 73 |
| Liraglutide | Sitagliptin | 2 | 0.14 | [-1.98, 2.26] | 0 |
| Exenatide | Placebo | 2 | -1.45 | [-4.56, 1.67] | 0 |
| Empagliflozin | Placebo | 4 | -4.35 | [-6.87, -1.82] | 83 |
| **Visceral adipose tissue reduction (Drug Classes)** | | | | | |
| GLP-1 R agonists | Placebo | 4 | -23.40 | [-41.41, -5.39] | 82 |
| GLP-1 R agonists | Basal Insulin | 3 | -30.97 | [-39.78, -22.16] | 0 |
| **Subcutaneous adipose tissue reduction (Drug Classes) (Drug Classes)** | | | | | |
| GLP-1 R agonists | Placebo | 4 | -27.38 | [-38.02, -16.74] | 0 |
| GLP-1 R agonists | Basal Insulin | 3 | -30.89 | [-51.44, -10.35] | 56 |
| DPP-4=Dipeptidyl peptidase-4. GLP-1=Glucagon-like peptide-1. GIP=Glucose-dependent insulinotropic polypeptide. SGLT-2=Sodium-glucose cotransporter-2. RA=Receptor agonist. MD=mean difference | | | | | |

| **Table S9:** P-scores ranking for Change in Liver Fat Content for drug classes | |
| --- | --- |
| **Drug class** | **P-score** |
| GIP/GLP-1 RA | 0.8771 |
| SGLT-2 inhibitor | 0.8439 |
| GLP-1 RA | 0.7170 |
| DPP-4 inhibitor | 0.5441 |
| Basal insulin | 0.4113 |
| Placebo | 0.3543 |
| Sulphonylurea | 0.3473 |
| Pioglitazone | 0.3059 |
| Metformin | 0.0991 |
| Treatments were ranked by means of P-scores. P-scores values range between 0 and 1, where a value of 1 means that a treatment ranks always best and a value of 0 means that a treatment ranks always worst. DPP-4=Dipeptidyl peptidase-4. GLP-1=Glucagon-like peptide-1. GIP=Glucose-dependent insulinotropic polypeptide. SGLT-2=Sodium-glucose cotransporter-2. RA=Receptor agonist. | |

| **Table S10:** Network meta-analysis results for liver fat content reduction (Agents) | | | | | | | | | | | | |
| --- | --- | --- | --- | --- | --- | --- | --- | --- | --- | --- | --- | --- |
| Empagliflozin |  |  |  |  |  |  |  |  |  |  |  |  |
| -0.78 [ -5.28; 3.71] | Exenatide |  |  |  |  |  |  |  |  |  |  |  |
| -0.85 [ -6.99; 5.29] | -0.07 [ -6.90; 6.77] | Dulaglutide |  |  |  |  |  |  |  |  |  |  |
| -2.15 [ -8.01; 3.71] | -1.37 [ -7.96; 5.22] | -1.30 [ -9.08; 6.48] | Canagliflozin |  |  |  |  |  |  |  |  |  |
| -2.35 [ -7.87; 3.17] | -1.57 [ -7.86; 4.72] | -1.50 [ -9.03; 6.03] | -0.20 [ -7.50; 7.10] | Vildagliptin |  |  |  |  |  |  |  |  |
| -2.63 [ -5.65; 0.39] | -1.85 [ -5.96; 2.27] | -1.78 [ -7.79; 4.23] | -0.48 [ -6.21; 5.24] | -0.28 [ -5.66; 5.10] | Liraglutide |  |  |  |  |  |  |  |
| **-3.31 [ -6.49; -0.13]** | -2.52 [ -7.16; 2.11] | -2.46 [ -8.79; 3.87] | -1.16 [ -7.22; 4.90] | -0.96 [ -6.69; 4.77] | -0.68 [ -3.66; 2.31] | Sitagliptin |  |  |  |  |  |  |
| -3.50 [ -7.94; 0.94] | -2.71 [ -8.08; 2.66] | -2.65 [ -9.43; 4.13] | -1.35 [ -7.88; 5.18] | -1.15 [ -7.37; 5.08] | -0.87 [ -5.13; 3.40] | -0.19 [ -4.89; 4.52] | Dapagliflozin |  |  |  |  |  |
| **-4.21 [ -7.95; -0.47]** | -3.43 [ -7.77; 0.92] | -3.36 [ -9.78; 3.06] | -2.06 [ -8.21; 4.09] | -1.86 [ -7.69; 3.97] | -1.58 [ -4.42; 1.26] | -0.90 [ -4.49; 2.69] | -0.71 [ -5.54; 4.11] | Glargine insulin |  |  |  |  |
| **-5.78 [-11.21; -0.34]** | -4.99 [-10.77; 0.78] | -4.93 [-12.44; 2.59] | -3.63 [-10.91; 3.66] | -3.43 [-10.45; 3.59] | -3.14 [ -7.75; 1.46] | -2.47 [ -7.90; 2.97] | -2.28 [ -8.49; 3.93] | -1.57 [ -6.90; 3.76] | Pioglitazone |  |  |  |
| **-7.41 [-25.34; 10.52]** | -6.62 [-24.87; 11.62] | -6.56 [-25.31; 12.19] | -5.26 [-23.92; 13.40] | -5.06 [-23.62; 13.50] | -4.78 [-22.68; 13.12] | -4.10 [-21.75; 13.55] | -3.91 [-22.18; 14.35] | -3.20 [-21.21; 14.81] | -1.63 [-20.10; 16.84] | Glimepiride |  |  |
| **-9.14 [-16.61; -1.67]** | **-8.35 [-16.07; -0.63]** | -8.29 [-17.38; 0.81] | -6.99 [-15.90; 1.92] | -6.79 [-15.48; 1.90] | -6.50 [-13.40; 0.39] | -5.83 [-13.30; 1.64] | -5.64 [-13.69; 2.41] | -4.93 [-12.32; 2.47] | -3.36 [ -8.48; 1.76] | -1.73 [-20.89; 17.44] | Metformin | . |
| **-4.35 [ -6.74; -1.96]** | -3.57 [ -7.42; 0.28] | -3.50 [ -9.15; 2.15] | -2.20 [ -7.55; 3.15] | -2.00 [ -6.97; 2.97] | -1.72 [ -3.76; 0.33] | -1.04 [ -3.89; 1.81] | -0.85 [ -4.60; 2.89] | -0.14 [ -3.19; 2.91] | 1.43 [ -3.53; 6.38] | 3.06 [-14.82; 20.94] | 4.79 [ -2.34; 11.91] | Placebo |
| Data are mean differences (95% CIs) of the column-deﬁning treatment compared with the row-deﬁning treatment. negative values favor the column-deﬁning treatment and positive values favor the row-defining treatment. For direct comparisons, Negative values favor the row-deﬁning treatment and positive values favor the column-defining treatment. Significant results are in bold. | | | | | | | | | | | | |

| **Table S11:** P-scores ranking for Change in Liver Fat Content for individual agents | |
| --- | --- |
| **Agent** | **P-score** |
| Empagliflozin | 0.8690 |
| Exenatide | 0.7774 |
| Dulaglutide | 0.7326 |
| Canagliflozin | 0.6130 |
| Vildagliptin | 0.5951 |
| Liraglutide | 0.5935 |
| Sitagliptin | 0.4875 |
| Dapagliflozin | 0.4658 |
| Glargine insulin | 0.3649 |
| Glimepiride | 0.3314 |
| Placebo | 0.3252 |
| Pioglitazone | 0.2540 |
| Metformin | 0.0905 |
| Treatments were ranked by means of P-scores. P-scores values range between 0 and 1, where a value of 1 means that a treatment ranks always best and a value of 0 means that a treatment ranks always worst. | |

| **Figure S2**: Network plot for Visceral Adipose Tissue |
| --- |
| 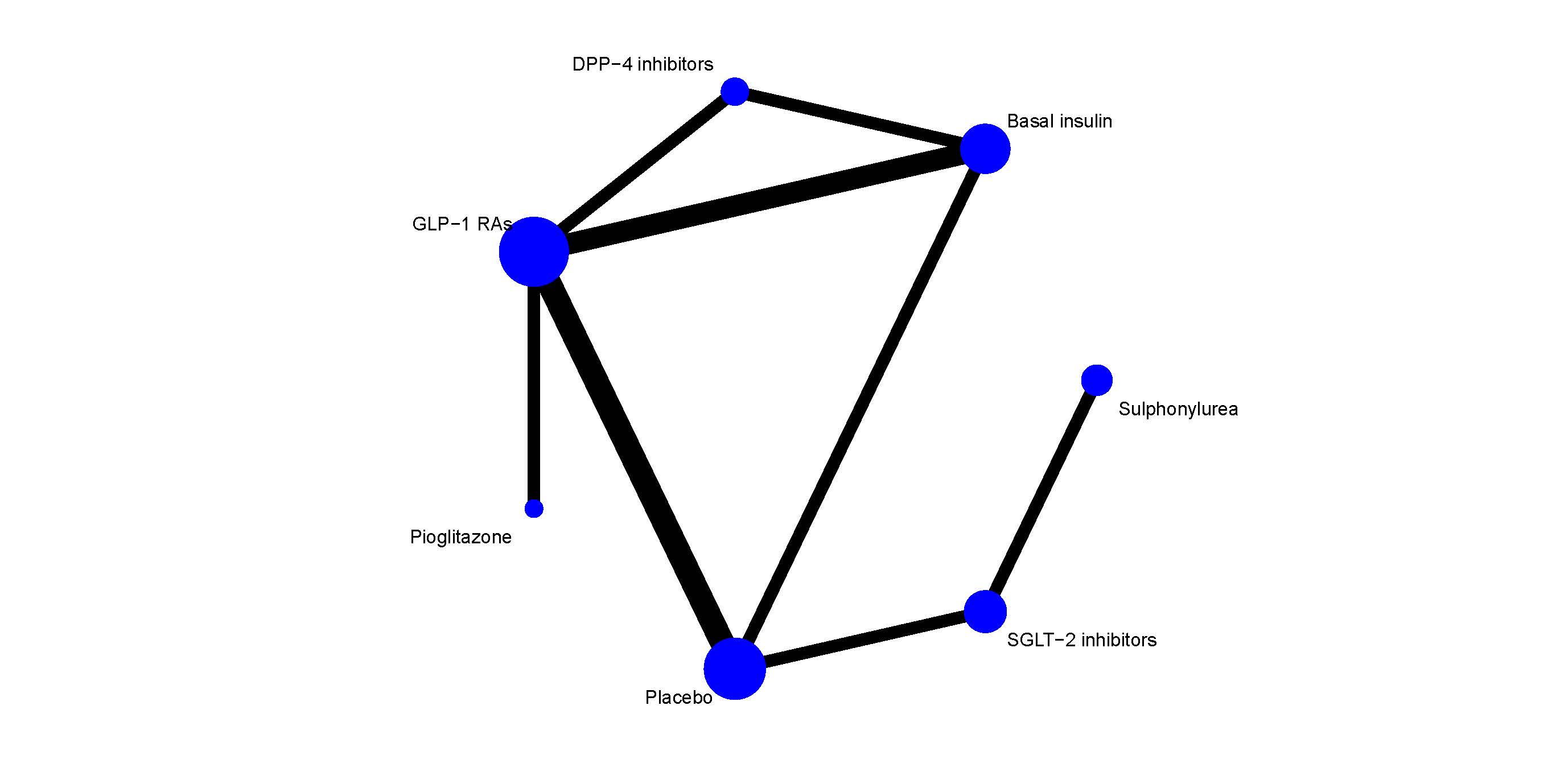 |
| Each circle indicates a treatment node. Lines connecting 2 nodes represent direct comparisons between 2 treatments. The size of the nodes is proportional to the number of trials evaluating each treatment. The thickness of the lines is proportional to the number of trials directly comparing the 2 connected treatments. DPP-4=dipeptidyl peptidase-4. GLP-1=Glucagon-like peptide-1. SGLT-2=Sodium-glucose cotransporter-2. |

| **Figure S3**: Network plot for Subcutaneous Adipose Tissue |
| --- |
| 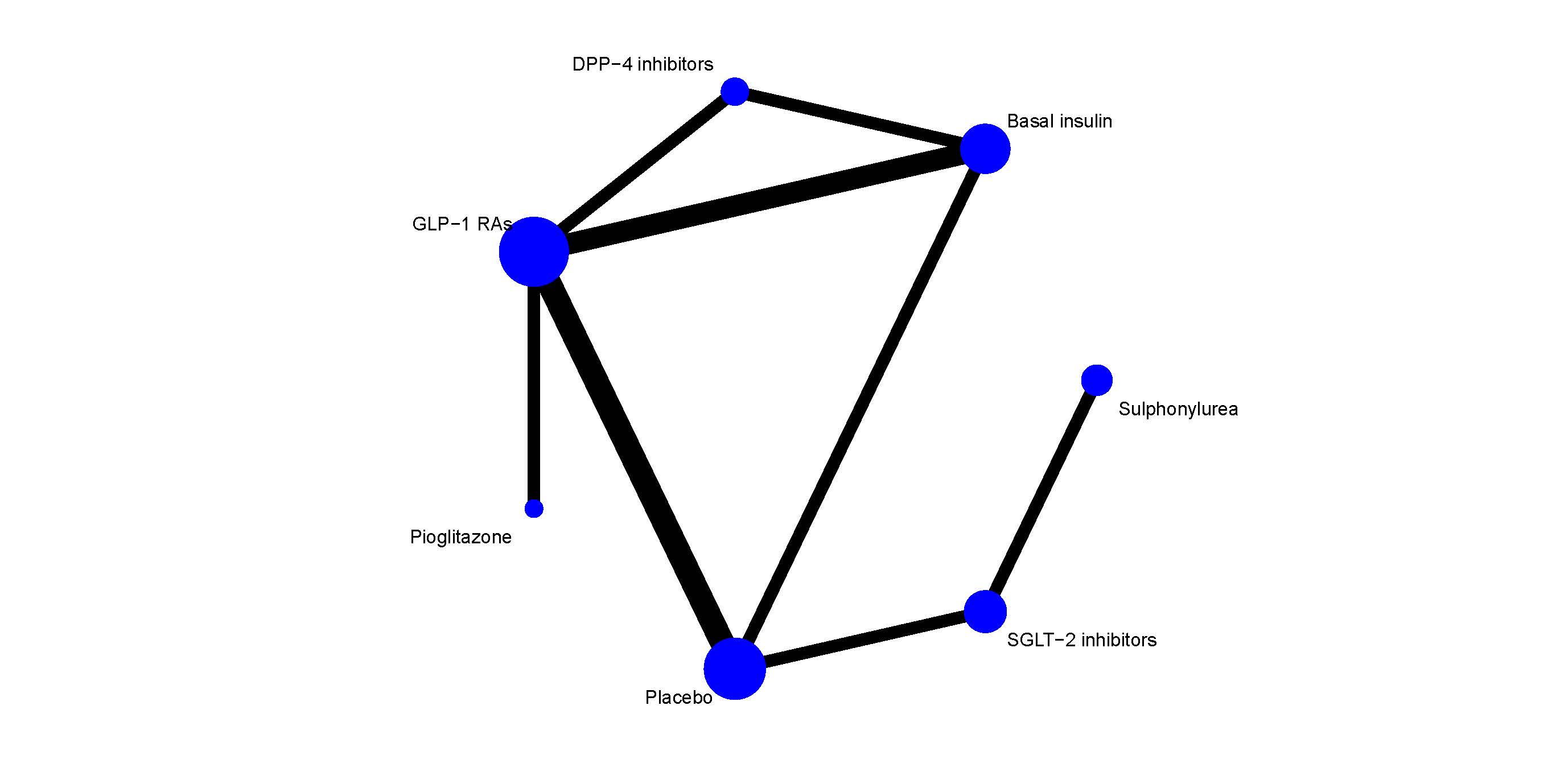 |
| Each circle indicates a treatment node. Lines connecting 2 nodes represent direct comparisons between 2 treatments. The size of the nodes is proportional to the number of trials evaluating each treatment. The thickness of the lines is proportional to the number of trials directly comparing the 2 connected treatments. DPP-4=dipeptidyl peptidase-4. GLP-1=Glucagon-like peptide-1. SGLT-2=Sodium-glucose cotransporter-2. |

| **Table S12:** Network meta-analysis results for Visceral Adipose Tissue reduction | | | | | | |
| --- | --- | --- | --- | --- | --- | --- |
| SGLT-2 inhibitors |  |  |  |  |  |  |
| 9.30 [-23.30; 41.89] | GLP-1 RAs |  |  |  |  |  |
| 3.57 [-37.43; 44.57] | -5.73 [-31.54; 20.08] | DPP-4 inhibitors |  |  |  |  |
| -17.58 [-52.89; 17.73] | -26.88 [-43.92; -9.83] | -21.15 [-47.55; 5.25] | Basal insulin |  |  |  |
| -13.20 [-64.77; 38.36] | -22.50 [-62.46; 17.46] | -16.77 [-64.34; 30.80] | 4.38 [-39.06; 47.82] | Pioglitazone |  |  |
| -22.20 [-51.18; 6.78] | -31.50 [-75.11; 12.12] | -25.77 [-75.98; 24.44] | -4.62 [-50.30; 41.06] | -9.00 [-68.15; 50.15] | Sulphonylurea |  |
| -15.70 [-44.81; 13.41] | **-25.00 [-39.66; -10.33]** | -19.27 [-48.15; 9.61] | 1.88 [-18.11; 21.87] | -2.50 [-45.06; 40.07] | 6.50 [-34.57; 47.57] | Placebo |
| Data are mean differences (95% CIs) of the column-deﬁning treatment compared with the row-deﬁning treatment. Negative values favor the column-deﬁning treatment and positive values favor the row-defining treatment. Significant results are in bold. DPP-4=Dipeptidyl peptidase-4. GLP-1=Glucagon-like peptide-1. SGLT-2=Sodium-glucose cotransporter-2. RA=Receptor agonist. | | | | | | |

| **Table S13:** Network meta-analysis results for Subcutaneous Adipose Tissue reduction | | | | | | |
| --- | --- | --- | --- | --- | --- | --- |
| SGLT-2 inhibitors |  |  |  |  |  |  |
| 14.06 [-16.30; 44.43] | GLP-1 RAs |  |  |  |  |  |
| 5.71 [-32.13; 43.55] | -8.35 [-32.14; 15.43] | DPP-4 inhibitors |  |  |  |  |
| -15.77 [-49.01; 17.47] | **-29.83 [-46.36; -13.31]** | -21.48 [-43.79; 0.83] | Basal insulin |  |  |  |
| 1.75 [-40.07; 43.57] | -12.31 [-41.07; 16.45] | -3.96 [-41.28; 33.36] | 17.52 [-15.65; 50.69] | Pioglitazone |  |  |
| **-40.00 [-65.49; -14.51]** | **-54.06 [-93.71; -14.42]** | **-45.71 [-91.33; -0.09]** | -24.23 [-66.12; 17.66] | -41.75 [-90.73; 7.22] | Sulphonylurea |  |
| -16.91 [-44.01; 10.19] | **-30.97 [-44.67; -17.28]** | -22.62 [-49.02; 3.78] | -1.14 [-20.39; 18.11] | -18.66 [-50.52; 13.19] | 23.09 [-14.11; 60.29] | Placebo |
| Data are mean differences (95% CIs) of the column-deﬁning treatment compared with the row-deﬁning treatment. Negative values favor the column-deﬁning treatment and positive values favor the row-defining treatment. Significant results are in bold. DPP-4=Dipeptidyl peptidase-4. GLP-1=Glucagon-like peptide-1. SGLT-2=Sodium-glucose cotransporter-2. RA=Receptor agonist. | | | | | | |

| **Table S14:** P-scores ranking for Change in Visceral Adipose Tissue | |
| --- | --- |
| **Drug class** | **P-score** |
| GLP-1 RA | 0.8609 |
| DPP-4 inhibitor | 0.7240 |
| SGLT-2 inhibitor | 0.6727 |
| Pioglitazone | 0.4048 |
| Placebo | 0.3150 |
| Basal insulin | 0.2752 |
| Sulphonylurea | 0.2475 |
| Treatments were ranked by means of P-scores. P-scores values range between 0 and 1, where a value of 1 means that a treatment ranks always best and a value of 0 means that a treatment ranks always worst. DPP-4=Dipeptidyl peptidase-4. GLP-1=Glucagon-like peptide-1. SGLT-2=Sodium-glucose cotransporter-2. RA=Receptor agonist. | |

| **Table S15:** P-scores ranking for Change in Subcutaneous Adipose Tissue | |
| --- | --- |
| **Drug class** | **P-score** |
| GLP-1 RA | 0.8946 |
| DPP-4 inhibitor | 0.7239 |
| Pioglitazone | 0.6380 |
| SGLT-2 inhibitor | 0.6242 |
| Basal insulin | 0.2957 |
| Placebo | 0.2708 |
| Sulphonylurea | 0.0529 |
| Treatments were ranked by means of P-scores. P-scores values range between 0 and 1, where a value of 1 means that a treatment ranks always best and a value of 0 means that a treatment ranks always worst. DPP-4=Dipeptidyl peptidase-4. GLP-1=Glucagon-like peptide-1. SGLT-2=Sodium-glucose cotransporter-2. RA=Receptor agonist. | |

| **Table S16**: Sensitivity analysis including trials that recruited drug naïve or metformin monotherapy treated patients | | | | | | |
| --- | --- | --- | --- | --- | --- | --- |
| SGLT-2 inhibitors |  |  |  |  |  |  |
| 3.26 [ -0.77; 7.28] | GLP-1 RAs |  |  |  |  |  |
| 2.07 [ -2.31; 6.44] | -1.19 [ -4.46; 2.08] | DPP-4 inhibitors |  |  |  |  |
| -0.10 [ -4.52; 4.31] | -3.36 [ -6.17; -0.55] | -2.17 [ -5.71; 1.38] | Basal insulin |  |  |  |
| 0.10 [ -5.41; 5.61] | -3.15 [ -6.92; 0.61] | -1.96 [ -6.95; 3.03] | 0.21 [ -4.49; 4.90] | Pioglitazone |  |  |
| -2.03 [-19.98; 15.91] | -5.29 [-23.00; 12.42] | -4.10 [-21.51; 13.31] | -1.93 [-19.70; 15.83] | -2.14 [-20.24; 15.97] | Sulphonylurea |  |
| -0.86 [ -3.94; 2.21] | **-4.12 [ -6.71; -1.52]** | -2.93 [ -6.04; 0.18] | -0.76 [ -3.93; 2.41] | -0.97 [ -5.54; 3.61] | 1.17 [-16.51; 18.85] | Placebo |
| Data are mean differences (95% CIs) of the column-deﬁning treatment compared with the row-deﬁning treatment. Negative values favor the column-deﬁning treatment and positive values favor the row-defining treatment. Significant results are in bold. DPP-4=Dipeptidyl peptidase-4. GLP-1=Glucagon-like peptide-1. SGLT-2=Sodium-glucose cotransporter-2. RA=Receptor agonist. | | | | | | |

| **Table S17**: Sensitivity analysis including trials that recruited patients with T2D and NAFLD | | | | | | |
| --- | --- | --- | --- | --- | --- | --- |
| SGLT-2 inhibitors |  |  |  |  |  |  |
| 0.01 [ -3.64; 3.67] | GLP-1 RAs |  |  |  |  |  |
| 0.59 [ -5.60; 6.79] | 0.58 [ -5.04; 6.20] | GIP/GLP-1 RAs |  |  |  |  |
| -2.14 [ -5.56; 1.29] | -2.15 [ -5.82; 1.52] | -2.73 [ -8.85; 3.39] | DPP-4 inhibitors |  |  |  |
| -3.70 [ -7.75; 0.36] | **-3.71 [ -6.80; -0.61]** | -4.29 [ -8.98; 0.40] | -1.56 [ -5.48; 2.37] | Basal insulin |  |  |
| -2.49 [ -8.31; 3.33] | -2.50 [ -7.03; 2.03] | -3.08 [-10.30; 4.14] | -0.35 [ -6.18; 5.48] | 1.21 [ -4.28; 6.69] | Pioglitazone |  |
| **-4.52 [ -7.06; -1.99]** | **-4.54 [ -7.56; -1.51]** | -5.12 [-11.01; 0.77] | -2.39 [ -5.88; 1.10] | -0.83 [ -4.38; 2.73] | -2.04 [ -7.48; 3.41] | Placebo |
| Data are mean differences (95% CIs) of the column-deﬁning treatment compared with the row-deﬁning treatment. Negative values favor the column-deﬁning treatment and positive values favor the row-defining treatment. Significant results are in bold. DPP-4=Dipeptidyl peptidase-4. GLP-1=Glucagon-like peptide-1. SGLT-2=Sodium-glucose cotransporter-2. RA=Receptor agonist. | | | | | | |

| **Table S18**: Sensitivity analysis including trials that at low risk of bias | | | | | |
| --- | --- | --- | --- | --- | --- |
| SGLT-2 inhibitors |  |  |  |  |  |
| -2.69 [ -6.81; 1.43] | GLP-1 RAs |  |  |  |  |
| -2.65 [ -8.35; 3.05] | 0.04 [ -3.96; 4.04] | GIP/GLP-1 RAs |  |  |  |
| -3.58 [ -8.57; 1.40] | -0.89 [ -3.89; 2.11] | -0.93 [ -5.18; 3.31] | DPP-4 inhibitors |  |  |
| **-6.94 [-11.97; -1.92]** | **-4.25 [ -7.20; -1.30]** | -4.29 [ -6.99; -1.59] | -3.36 [ -6.63; -0.08] | Basal insulin |  |
| **-5.53 [ -9.06; -2.00]** | **-2.84 [ -4.96; -0.72]** | -2.88 [ -7.35; 1.60] | -1.95 [ -5.46; 1.57] | 1.41 [ -2.16; 4.98] | Placebo |
| Data are mean differences (95% CIs) of the column-deﬁning treatment compared with the row-deﬁning treatment. Negative values favor the column-deﬁning treatment and positive values favor the row-defining treatment. Significant results are in bold. DPP-4=Dipeptidyl peptidase-4. GLP-1=Glucagon-like peptide-1. SGLT-2=Sodium-glucose cotransporter-2. RA=Receptor agonist. | | | | | |

| **Table S19**: Sensitivity analysis including trials without imputations | | | | | | |
| --- | --- | --- | --- | --- | --- | --- |
| SGLT-2 inhibitors |  |  |  |  |  |  |
| -0.02 [ -3.80; 3.75] | GLP-1 RAs |  |  |  |  |  |
| 1.12 [ -6.16; 8.40] | 1.14 [ -5.49; 7.77] | GIP/GLP-1 RAs |  |  |  |  |
| -1.69 [ -5.92; 2.54] | -1.67 [ -5.98; 2.65] | -2.81 [-10.15; 4.54] | DPP-4 inhibitors |  |  |  |
| -5.79 [-24.10; 12.52] | -5.77 [-24.10; 12.57] | -6.91 [-26.18; 12.36] | -4.10 [-21.92; 13.72] | Sulphonylurea |  |  |
| -3.17 [ -7.77; 1.43] | -3.15 [ -6.64; 0.35] | -4.29 [ -9.93; 1.35] | -1.48 [ -6.19; 3.23] | 2.62 [-15.81; 21.05] | Basal insulin |  |
| -1.92 [ -5.03; 1.19] | -1.90 [ -4.44; 0.64] | -3.04 [ -9.86; 3.78] | -0.23 [ -4.57; 4.10] | 3.87 [-14.47; 22.21] | 1.25 [ -2.58; 5.08] | Placebo |
| Data are mean differences (95% CIs) of the column-deﬁning treatment compared with the row-deﬁning treatment. Negative values favor the column-deﬁning treatment and positive values favor the row-defining treatment. Significant results are in bold. DPP-4=Dipeptidyl peptidase-4. GLP-1=Glucagon-like peptide-1. SGLT-2=Sodium-glucose cotransporter-2. RA=Receptor agonist. | | | | | | |

| **Table S20**: Evaluation of heterogeneity in network meta-analyses | | |
| --- | --- | --- |
| **Outcome** | **Estimated τ^2^** | **Predictive distributions of τ^2 (^median, 95% range)** |
| **Steatosis-related Outcomes** | | |
| Absolute Change in LFC (Drug Classes) | 4.7 | 0.027 (0.00001-4.95) |
| Absolute Change in LFC (Agents) | 5.8 | 0.027 (0.00001-4.95) |
| VAT reduction | 166.3 | 0.027 (0.00001-4.95) |
| SAT reduction | 83.7 | 0.027 (0.00001-4.95) |
| LFC: Liver fat content, VAT: Visceral Adipose Tissue, SAT: Subcutaneous Adipose Tissue | | |

| **Table S21**: Evaluation of inconsistency in network meta-analyses | | | |
| --- | --- | --- | --- |
| **Outcome** | **Design-by-treatment interaction model** | | |
|  | Q | df | p-value |
| Absolute Change in LFC (Drug Classes) | 5.4 | 7 | 0.6118 |
| Absolute Change in LFC (Agents) | 10.4 | 9 | 0.3176 |
| VAT reduction | 16.6 | 3 | 0.0008 |
| SAT reduction | 3.9 | 3 | 0.2704 |
| LFC: Liver fat content, VAT: Visceral Adipose Tissue, SAT: Subcutaneous Adipose Tissue | | | |

| **Figure S4:** Comparison adjusted funnel plot for change from baseline Liver Fat Content (Drug Classes) |
| --- |
| 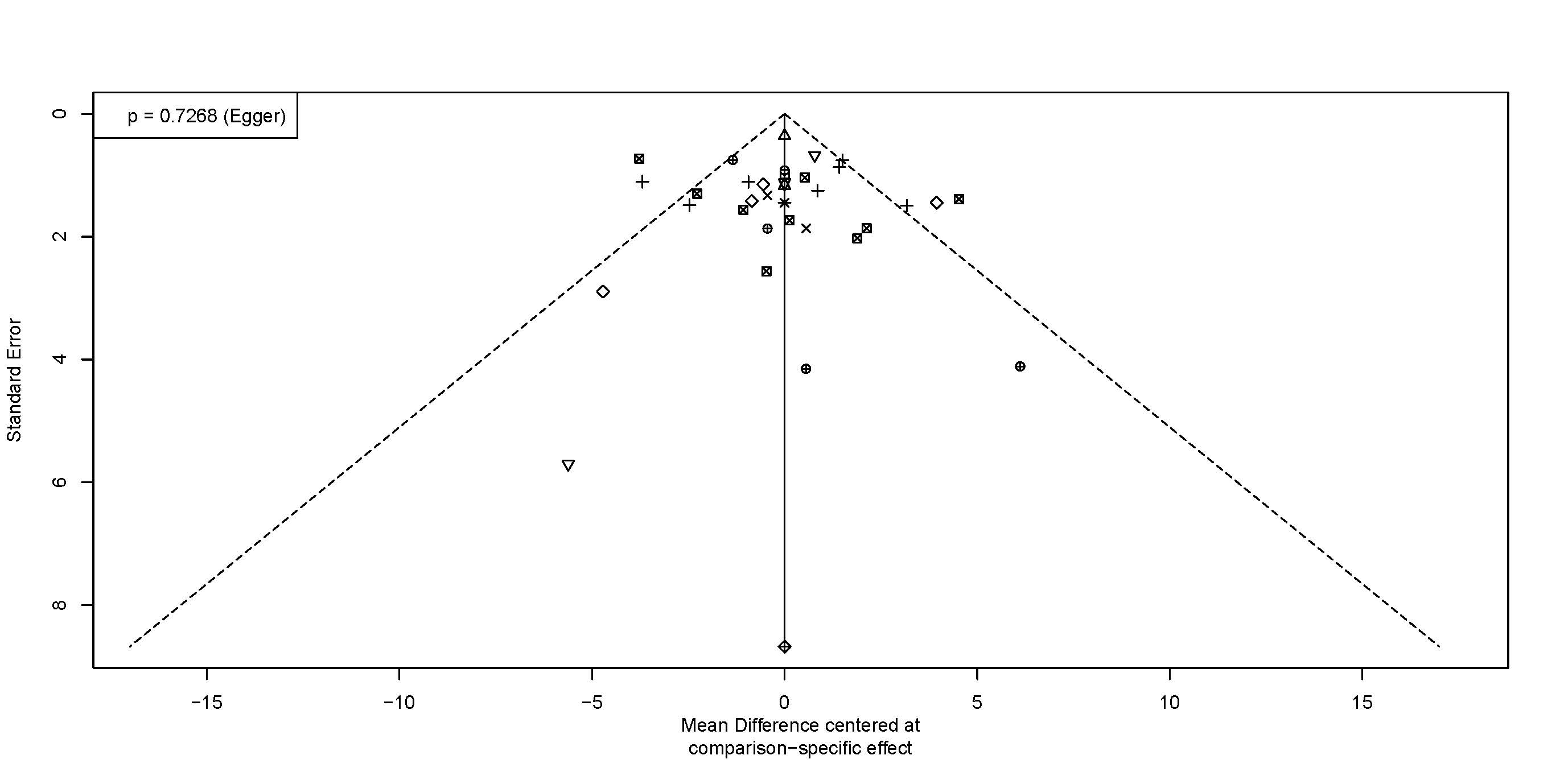 |

| **Figure S4:** Comparison adjusted funnel plot for change from baseline Liver Fat Content (Agents) |
| --- |
| 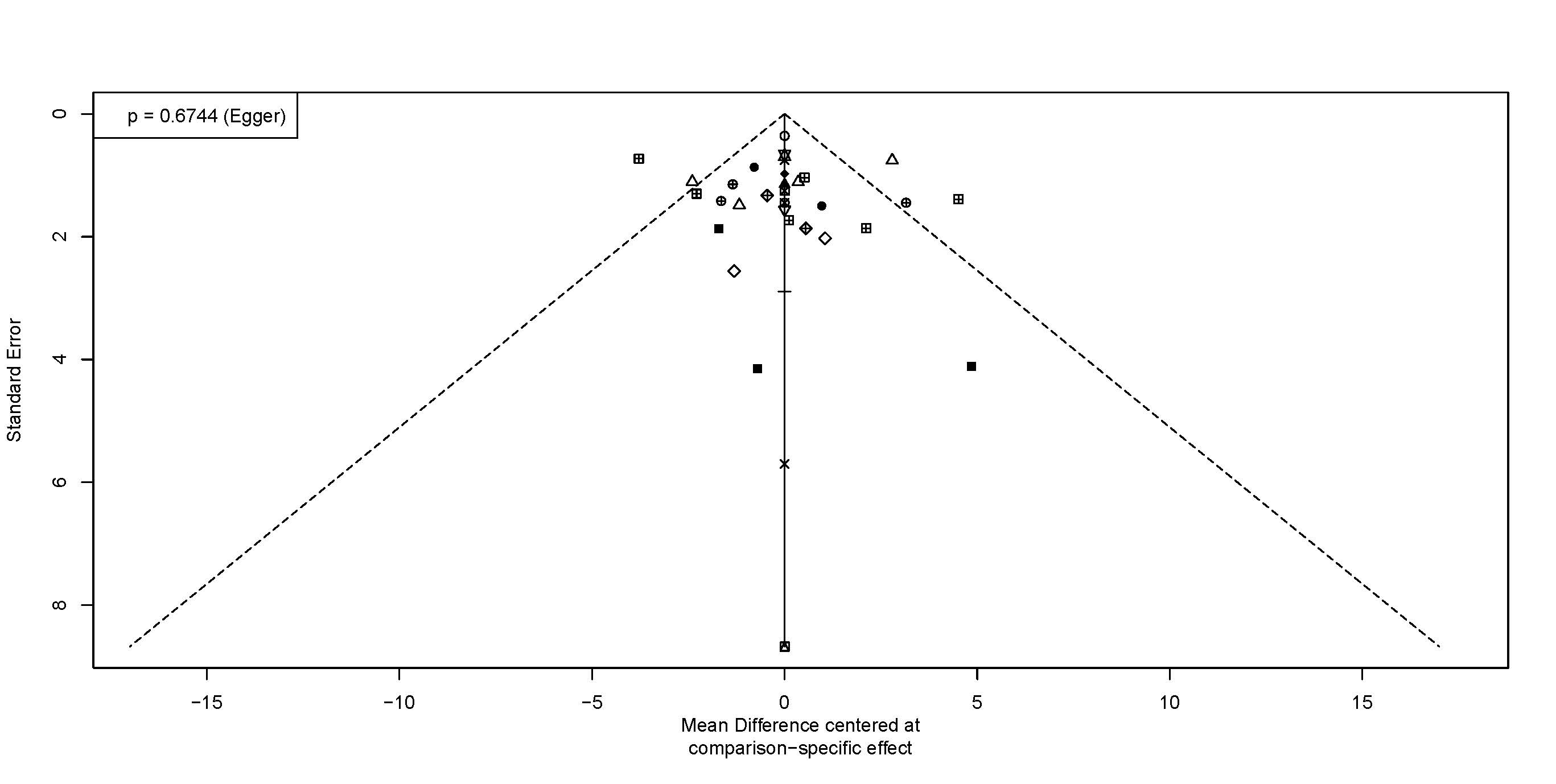 |

| **Table S22**: Confidence in effects estimates for the primary outcome (Drug classes) | | | | | | | | |
| --- | --- | --- | --- | --- | --- | --- | --- | --- |
| Comparison | Number of studies | Within-study bias | Reporting bias | Indirectness | Imprecision | Heterogeneity | Incoherence | Confidence rating |
| Basal insulin: DPP-4 inhibitors | 1 | Some concerns | Low risk | No concerns | No concerns | Some concerns | No concerns | Low |
| Basal insulin:GIP/GLP-1 RAs | 1 | No concerns | Low risk | No concerns | Some concerns | No concerns | No concerns | Moderate |
| Basal insulin:GLP-1 RAs | 4 | Some concerns | Low risk | No concerns | No concerns | Some concerns | No concerns | Low |
| Basal insulin:Placebo | 1 | Some concerns | Low risk | No concerns | No concerns | Major concerns | No concerns | Very low |
| DPP-4 inhibitors:GLP-1 RAs | 2 | Some concerns | Low risk | No concerns | No concerns | Some concerns | No concerns | Low |
| DPP-4 inhibitors:Placebo | 4 | Some concerns | Low risk | No concerns | No concerns | Some concerns | No concerns | Low |
| DPP-4 inhibitors:SGLT-2 inhibitors | 1 | Major concerns | Low risk | No concerns | No concerns | Some concerns | No concerns | Very low |
| DPP-4 inhibitors:Sulphonylurea | 1 | Major concerns | Low risk | No concerns | Major concerns | No concerns | No concerns | Very low |
| GLP-1 RAs:Pioglitazone | 2 | Major concerns | Low risk | No concerns | Some concerns | No concerns | No concerns | Very low |
| GLP-1 RAs:Placebo | 9 | Some concerns | Low risk | No concerns | No concerns | Some concerns | No concerns | Low |
| Metformin:Pioglitazone | 1 | Major concerns | Low risk | No concerns | Some concerns | No concerns | No concerns | Very low |
| Placebo:SGLT-2 inhibitors | 7 | Some concerns | Low risk | No concerns | No concerns | Some concerns | No concerns | Low |
| Basal insulin:Metformin | 0 | Major concerns | Low risk | No concerns | Some concerns | No concerns | No concerns | Very low |
| Basal insulin:Pioglitazone | 0 | Major concerns | Low risk | No concerns | Some concerns | Some concerns | No concerns | Very low |
| Basal insulin:SGLT-2 inhibitors | 0 | Some concerns | Low risk | No concerns | Some concerns | No concerns | No concerns | Low |
| Basal insulin:Sulphonylurea | 0 | Some concerns | Low risk | No concerns | Major concerns | No concerns | No concerns | Very low |
| DPP-4 inhibitors:GIP/GLP-1 RAs | 0 | Some concerns | Low risk | No concerns | Some concerns | No concerns | No concerns | Low |
| DPP-4 inhibitors:Metformin | 0 | Major concerns | Low risk | No concerns | Some concerns | No concerns | No concerns | Very low |
| DPP-4 inhibitors:Pioglitazone | 0 | Some concerns | Low risk | No concerns | Some concerns | No concerns | No concerns | Low |
| GIP/GLP-1 RAs:GLP-1 RAs | 0 | Some concerns | Low risk | No concerns | Some concerns | No concerns | No concerns | Low |
| GIP/GLP-1 RAs:Metformin | 0 | Some concerns | Low risk | No concerns | No concerns | Some concerns | No concerns | Low |
| GIP/GLP-1 RAs:Pioglitazone | 0 | Some concerns | Low risk | No concerns | Some concerns | No concerns | No concerns | Low |
| GIP/GLP-1 RAs:Placebo | 0 | Some concerns | Low risk | No concerns | Some concerns | No concerns | No concerns | Low |
| GIP/GLP-1 RAs:SGLT-2 inhibitors | 0 | Some concerns | Low risk | No concerns | Some concerns | Some concerns | No concerns | Low |
| GIP/GLP-1 RAs:Sulphonylurea | 0 | Some concerns | Low risk | No concerns | Major concerns | No concerns | No concerns | Very low |
| GLP-1 RAs:Metformin | 0 | Major concerns | Low risk | No concerns | No concerns | Some concerns | No concerns | Very low |
| GLP-1 RAs:SGLT-2 inhibitors | 0 | Some concerns | Low risk | No concerns | No concerns | Some concerns | No concerns | Low |
| GLP-1 RAs:Sulphonylurea | 0 | Some concerns | Low risk | No concerns | Major concerns | No concerns | No concerns | Very low |
| Metformin:Placebo | 0 | Major concerns | Low risk | No concerns | Some concerns | No concerns | No concerns | Very low |
| Metformin:SGLT-2 inhibitors | 0 | Major concerns | Low risk | No concerns | No concerns | Some concerns | No concerns | Very low |
| Metformin:Sulphonylurea | 0 | Major concerns | Low risk | No concerns | Major concerns | No concerns | No concerns | Very low |
| Pioglitazone:Placebo | 0 | Major concerns | Low risk | No concerns | Some concerns | Some concerns | No concerns | Very low |
| Pioglitazone:SGLT-2 inhibitors | 0 | Major concerns | Low risk | No concerns | Some concerns | No concerns | No concerns | Very low |
| Pioglitazone:Sulphonylurea | 0 | Major concerns | Low risk | No concerns | Major concerns | No concerns | No concerns | Very low |
| Placebo:Sulphonylurea | 0 | Major concerns | Low risk | No concerns | Major concerns | No concerns | No concerns | Very low |
| SGLT-2 inhibitors:Sulphonylurea | 0 | Major concerns | Low risk | No concerns | Major concerns | No concerns | No concerns | Very low |
| DPP-4=Dipeptidyl peptidase-4. GLP-1=Glucagon-like peptide-1. SGLT-2=Sodium-glucose cotransporter-2. RA=Receptor agonist. | | | | | | | | |

| **Table S23**: Confidence in effects estimates for the primary outcome (Agents) | | | | | | | | |
| --- | --- | --- | --- | --- | --- | --- | --- | --- |
| Comparison | Number of studies | Within-study bias | Reporting bias | Indirectness | Imprecision | Heterogeneity | Incoherence | Confidence rating |
| Canagliflozin:Placebo | 1 | Major concerns | Low risk | No concerns | Some concerns | Some concerns | No concerns | Very low |
| Dapagliflozin:Placebo | 2 | Major concerns | Low risk | No concerns | No concerns | Major concerns | No concerns | Very low |
| Dulaglutide:Placebo | 1 | Major concerns | Low risk | No concerns | Some concerns | No concerns | No concerns | Very low |
| Empagliflozin:Placebo | 4 | Some concerns | Low risk | No concerns | No concerns | Some concerns | No concerns | Low |
| Empagliflozin:Sitagliptin | 1 | Major concerns | Low risk | No concerns | No concerns | Some concerns | No concerns | Very low |
| Exenatide:Glargine insulin | 1 | Some concerns | Low risk | No concerns | Some concerns | No concerns | No concerns | Low |
| Exenatide:Pioglitazone | 1 | Some concerns | Low risk | No concerns | Some concerns | No concerns | No concerns | Low |
| Exenatide:Placebo | 2 | Some concerns | Low risk | No concerns | Some concerns | No concerns | No concerns | Low |
| Glargine insulin:Liraglutide | 3 | Some concerns | Low risk | No concerns | No concerns | Some concerns | No concerns | Low |
| Glargine insulin:Placebo | 1 | Some concerns | Low risk | No concerns | No concerns | Major concerns | No concerns | Very low |
| Glargine insulin:Sitagliptin | 1 | Some concerns | Low risk | No concerns | No concerns | Major concerns | No concerns | Very low |
| Glimepiride:Sitagliptin | 1 | Major concerns | Low risk | No concerns | Major concerns | No concerns | No concerns | Very low |
| Liraglutide:Pioglitazone | 1 | Major concerns | Low risk | No concerns | Some concerns | No concerns | No concerns | Very low |
| Liraglutide:Placebo | 6 | Some concerns | Low risk | No concerns | No concerns | Some concerns | No concerns | Low |
| Liraglutide:Sitagliptin | 2 | Some concerns | Low risk | No concerns | No concerns | Major concerns | No concerns | Very low |
| Metformin:Pioglitazone | 1 | Major concerns | Low risk | No concerns | Some concerns | No concerns | No concerns | Very low |
| Placebo:Sitagliptin | 3 | Some concerns | Low risk | No concerns | No concerns | Some concerns | No concerns | Low |
| Placebo:Vildagliptin | 1 | Major concerns | Low risk | No concerns | Some concerns | Some concerns | No concerns | Very low |
| Canagliflozin:Dapagliflozin | 0 | Major concerns | Low risk | No concerns | Major concerns | No concerns | No concerns | Very low |
| Canagliflozin:Dulaglutide | 0 | Major concerns | Low risk | No concerns | Major concerns | No concerns | No concerns | Very low |
| Canagliflozin:Empagliflozin | 0 | Major concerns | Low risk | No concerns | Some concerns | Some concerns | No concerns | Very low |
| Canagliflozin:Exenatide | 0 | Some concerns | Low risk | No concerns | Major concerns | No concerns | No concerns | Very low |
| Canagliflozin:Glargine insulin | 0 | Major concerns | Low risk | No concerns | Some concerns | Some concerns | No concerns | Very low |
| Canagliflozin:Glimepiride | 0 | Major concerns | Low risk | No concerns | Major concerns | No concerns | No concerns | Very low |
| Canagliflozin:Liraglutide | 0 | Major concerns | Low risk | No concerns | Major concerns | No concerns | No concerns | Very low |
| Canagliflozin:Metformin | 0 | Major concerns | Low risk | No concerns | Some concerns | No concerns | No concerns | Very low |
| Canagliflozin:Pioglitazone | 0 | Major concerns | Low risk | No concerns | Some concerns | Some concerns | No concerns | Very low |
| Canagliflozin:Sitagliptin | 0 | Major concerns | Low risk | No concerns | Some concerns | Some concerns | No concerns | Very low |
| Canagliflozin:Vildagliptin | 0 | Major concerns | Low risk | No concerns | Major concerns | No concerns | No concerns | Very low |
| Dapagliflozin:Dulaglutide | 0 | Major concerns | Low risk | No concerns | Some concerns | Some concerns | No concerns | Very low |
| Dapagliflozin:Empagliflozin | 0 | Some concerns | Low risk | No concerns | Some concerns | No concerns | No concerns | Very low |
| Dapagliflozin:Exenatide | 0 | Some concerns | Low risk | No concerns | Some concerns | Some concerns | No concerns | Low |
| Dapagliflozin:Glargine insulin | 0 | Some concerns | Low risk | No concerns | Some concerns | Some concerns | No concerns | Low |
| Dapagliflozin:Glimepiride | 0 | Major concerns | Low risk | No concerns | Major concerns | No concerns | No concerns | Very low |
| Dapagliflozin:Liraglutide | 0 | Some concerns | Low risk | No concerns | Some concerns | Some concerns | No concerns | Low |
| Dapagliflozin:Metformin | 0 | Major concerns | Low risk | No concerns | Some concerns | No concerns | No concerns | Very low |
| Dapagliflozin:Pioglitazone | 0 | Major concerns | Low risk | No concerns | Some concerns | Some concerns | No concerns | Very low |
| Dapagliflozin:Sitagliptin | 0 | Some concerns | Low risk | No concerns | No concerns | Major concerns | No concerns | Very low |
| Dapagliflozin:Vildagliptin | 0 | Major concerns | Low risk | No concerns | Major concerns | No concerns | No concerns | Very low |
| Dulaglutide:Empagliflozin | 0 | Major concerns | Low risk | No concerns | Major concerns | No concerns | No concerns | Very low |
| Dulaglutide:Exenatide | 0 | Some concerns | Low risk | No concerns | Major concerns | No concerns | No concerns | Very low |
| Dulaglutide:Glargine insulin | 0 | Major concerns | Low risk | No concerns | Some concerns | Some concerns | No concerns | Very low |
| Dulaglutide:Glimepiride | 0 | Major concerns | Low risk | No concerns | Major concerns | No concerns | No concerns | Very low |
| Dulaglutide:Liraglutide | 0 | Major concerns | Low risk | No concerns | Some concerns | Some concerns | No concerns | Very low |
| Dulaglutide:Metformin | 0 | Major concerns | Low risk | No concerns | Some concerns | No concerns | No concerns | Very low |
| Dulaglutide:Pioglitazone | 0 | Major concerns | Low risk | No concerns | Some concerns | No concerns | No concerns | Very low |
| Dulaglutide:Sitagliptin | 0 | Major concerns | Low risk | No concerns | Some concerns | Some concerns | No concerns | Very low |
| Dulaglutide:Vildagliptin | 0 | Major concerns | Low risk | No concerns | Major concerns | No concerns | No concerns | Very low |
| Empagliflozin:Exenatide | 0 | Some concerns | Low risk | No concerns | Some concerns | Some concerns | No concerns | Low |
| Empagliflozin:Glargine insulin | 0 | Some concerns | Low risk | No concerns | No concerns | Some concerns | No concerns | Low |
| Empagliflozin:Glimepiride | 0 | Major concerns | Low risk | No concerns | Major concerns | No concerns | No concerns | Very low |
| Empagliflozin:Liraglutide | 0 | Some concerns | Low risk | No concerns | Some concerns | No concerns | No concerns | Low |
| Empagliflozin:Metformin | 0 | Major concerns | Low risk | No concerns | No concerns | Some concerns | No concerns | Very low |
| Empagliflozin:Pioglitazone | 0 | Major concerns | Low risk | No concerns | No concerns | Some concerns | No concerns | Very low |
| Empagliflozin:Vildagliptin | 0 | Major concerns | Low risk | No concerns | Some concerns | Some concerns | No concerns | Very low |
| Exenatide:Glimepiride | 0 | Some concerns | Low risk | No concerns | Major concerns | No concerns | No concerns | Very low |
| Exenatide:Liraglutide | 0 | Some concerns | Low risk | No concerns | Some concerns | No concerns | No concerns | Low |
| Exenatide:Metformin | 0 | Major concerns | Low risk | No concerns | No concerns | Some concerns | No concerns | Very low |
| Exenatide:Sitagliptin | 0 | Some concerns | Low risk | No concerns | Some concerns | No concerns | No concerns | Low |
| Exenatide:Vildagliptin | 0 | Some concerns | Low risk | No concerns | Some concerns | Some concerns | No concerns | Low |
| Glargine insulin:Glimepiride | 0 | Some concerns | Low risk | No concerns | Major concerns | No concerns | No concerns | Very low |
| Glargine insulin:Metformin | 0 | Major concerns | Low risk | No concerns | Some concerns | No concerns | No concerns | Very low |
| Glargine insulin:Pioglitazone | 0 | Major concerns | Low risk | No concerns | Some concerns | Some concerns | No concerns | Very low |
| Glargine insulin:Vildagliptin | 0 | Major concerns | Low risk | No concerns | Some concerns | Some concerns | No concerns | Very low |
| Glimepiride:Liraglutide | 0 | Some concerns | Low risk | No concerns | Major concerns | No concerns | No concerns | Very low |
| Glimepiride:Metformin | 0 | Major concerns | Low risk | No concerns | Major concerns | No concerns | No concerns | Very low |
| Glimepiride:Pioglitazone | 0 | Major concerns | Low risk | No concerns | Major concerns | No concerns | No concerns | Very low |
| Glimepiride:Placebo | 0 | Major concerns | Low risk | No concerns | Major concerns | No concerns | No concerns | Very low |
| Glimepiride:Vildagliptin | 0 | Major concerns | Low risk | No concerns | Major concerns | No concerns | No concerns | Very low |
| Liraglutide:Metformin | 0 | Major concerns | Low risk | No concerns | Some concerns | No concerns | No concerns | Very low |
| Liraglutide:Vildagliptin | 0 | Major concerns | Low risk | No concerns | Major concerns | No concerns | No concerns | Very low |
| Metformin:Placebo | 0 | Major concerns | Low risk | No concerns | Some concerns | No concerns | No concerns | Very low |
| Metformin:Sitagliptin | 0 | Major concerns | Low risk | No concerns | Some concerns | No concerns | No concerns | Very low |
| Metformin:Vildagliptin | 0 | Major concerns | Low risk | No concerns | Some concerns | No concerns | No concerns | Very low |
| Pioglitazone:Placebo | 0 | Major concerns | Low risk | No concerns | Some concerns | Some concerns | No concerns | Very low |
| Pioglitazone:Sitagliptin | 0 | Some concerns | Low risk | No concerns | Some concerns | Some concerns | No concerns | Low |
| Pioglitazone:Vildagliptin | 0 | Major concerns | Low risk | No concerns | Some concerns | Some concerns | No concerns | Very low |
| Sitagliptin:Vildagliptin | 0 | Major concerns | Low risk | No concerns | Some concerns | Some concerns | No concerns | Very low |
